# Comparative analytical study of suction drum foundation penetration characteristics of guide frame platforms with real measurements

**DOI:** 10.1371/journal.pone.0299647

**Published:** 2024-02-29

**Authors:** Xin Zhang, Yuansong Li, Mingyue Liu, Shaoqi Ye, HuaQuan Liu

**Affiliations:** 1 School of Civil Engineering and Architecture, Wuhan Institute of Technology, Wuhan, 430074, Hubei, China; 2 The 5th Engineering Co., Ltd, China Railway Major Bridge Engineering, Jiujiang, 32000, Jiangxi China; Amirkabir University of Technology (Tehran Polytechnic), ISLAMIC REPUBLIC OF IRAN

## Abstract

A suction bucket foundation is a new type offering high construction efficiency, precise positioning, cost-effectiveness, and environmental friendliness. It has been extensively employed in marine resource development, particularly in offshore wind power and oil and gas extraction. It usually involves multiple suction bucket conduit rack platforms during offshore construction projects. Accurately predicting the sinking penetration resistance and determining the suction value is crucial during the construction of the suction bucket foundation, as it ensures the safe sinking of the platform foundation to the designated depth. This paper examines the feasibility of the suction bucket foundation’s sinking, sinking penetration resistance, suction value, and self-weight penetration depth, using the offshore wind farm guiding frame platform foundation project in Yangjiang, Guangdong Province, as a basis of analysis. The measured data is analyzed using the API specification static equilibrium analysis method, ABAQUS finite element analysis, and data mining techniques. The suction drum base platform’s sinking process was monitored for negative pressure and penetration resistance. These observed values were compared to theoretical and finite element calculations. Results demonstrated that the API specification’s theoretical calculations and finite element analyses effectively predict sinking penetration resistance, the suction force value, and the penetration depth for self-gravitational penetration. On-site engineering data fit these theoretical calculations, and finite element analyses well. The findings from this study have enriched the engineering application database of the suction drum foundation, providing a valuable reference for the design and construction of similar projects and establishing the groundwork for further promotion and application.

## 1. Introduction

The development of marine resources has become a strategic goal for countries worldwide since the 21st century started. The development and construction of marine resources require a safe and efficient auxiliary equipment platform. The suction drum foundation, a new type of offshore platform foundation, has an extensive application prospect and is highly favored by the marine engineering community [1–4].

The suction drum foundation is also referred to as suction pile, suction barrel, suction anchor, and caisson foundation, among other similar names. The suction barrel foundation utilizes a specialized construction process whereby the foundation is initially sunk through the self-weight of the structure. After that, the suction pump pumps seawater out of the barrel, creating a pressure difference between the inside and outside, causing the foundation to sink further to the desired depth. The suction drum foundation offers several advantages, including precise positioning, high construction efficiency, low cost, low noise output, simple recyclability, reusability, and lesser susceptibility to the effects of water depth.

The suction bucket foundation is frequently utilized in different types of offshore platform construction in China. However, its application in offshore wind power foundations is unprecedented. The depletion of fossil energy and the rise in carbon dioxide emissions make developing renewable energy a vital solution to this issue [5]. Offshore wind power, as a form of marine renewable energy, offers several benefits compared to onshore wind power. Not only does it not occupy land area, but it also generates low levels of noise pollution, leading to its rapid development in recent years. Unlike marine platform design, offshore wind power foundations must withstand cyclic loads from wind turbines and environmental loads for 25 years, with higher leveling requirements. As a result, the application of suction buckets in the offshore wind power foundation field faces more challenges and difficulties.

In recent years, global scholars researching wind power foundations have primarily focused on two main aspects: the superstructure and the lower foundation. The research conducted in the superstructure domain primarily centers on the monitoring and observation, structural performance, and seismic analysis of wind power foundations. Notably, successful outcomes have been achieved [6–17]. The research concerning the lower section of the foundation centers on the suction drum foundation’s penetrating and bearing characteristics [18–20].

The central components of studying the sinking characteristics are the accurate prediction of the sinking resistance and the appropriate application of the suction force value. Sinking resistance and the necessary suction force are primarily calculated using the following theoretical methods: ① based on soil properties (API, Houlsby, and Byrne), ② based on CPT data (DNV, Senders), and ③ based on a combination of soil properties and CPT data (Anderson), as evidenced by previous studies [21–29]. The studies mentioned above only focus on a single soil layer and lack verification from engineering examples. Both domestic and foreign researchers have few studies on the sinking resistance of suction drum foundations in mixed clay and sandy soil layers, even though most engineering projects involve mixed soil layers, such as powdery clay and sandy clay. The cited method provides a vital theoretical foundation for constructing a suction drum foundation. Nevertheless, no quantitative analysis method based on a model test and construction experience exists, and the critical parameters of the approach are slightly different from actual engineering data.

This paper uses the Guangdong Electricity Yangjiang Shapa offshore wind power project as a research case study. It combines the site investigation report and structural parameters of the suction drum foundation, applies technical methods, and combines theoretical analysis, numerical simulation, and data mining. It conducts a comparative analysis of the suction drum foundation’s sinking resistance and suction force. The results can serve as a foundation for constructing a suction drum conduit rack foundation, providing theoretical guidance and technical support for designing and constructing a suction drum foundation. Technological support. Successful implementation of the suction drum foundation on the guiding frame platform of the Yangjiang wind power project will enhance the suction drum foundation’s application database. It can serve as a reference for the design and construction of similar projects, laying the groundwork for further promoting and applying the suction drum foundation.

This paper presents several innovations. Firstly, the ALE method successfully simulates the suction bucket’s penetration process, revealing the penetration resistance and the bearing mechanism of the soil layer. Secondly, the measured data of engineering construction is compared and analyzed with the theoretical formulae to validate the theory’s applicability. Thirdly, the summary of the engineering cases, accumulating the original data for developing the theory of geotechnical penetration.

## 2. Engineering background

### 2.1. Overview of the project

**Fig 1:**
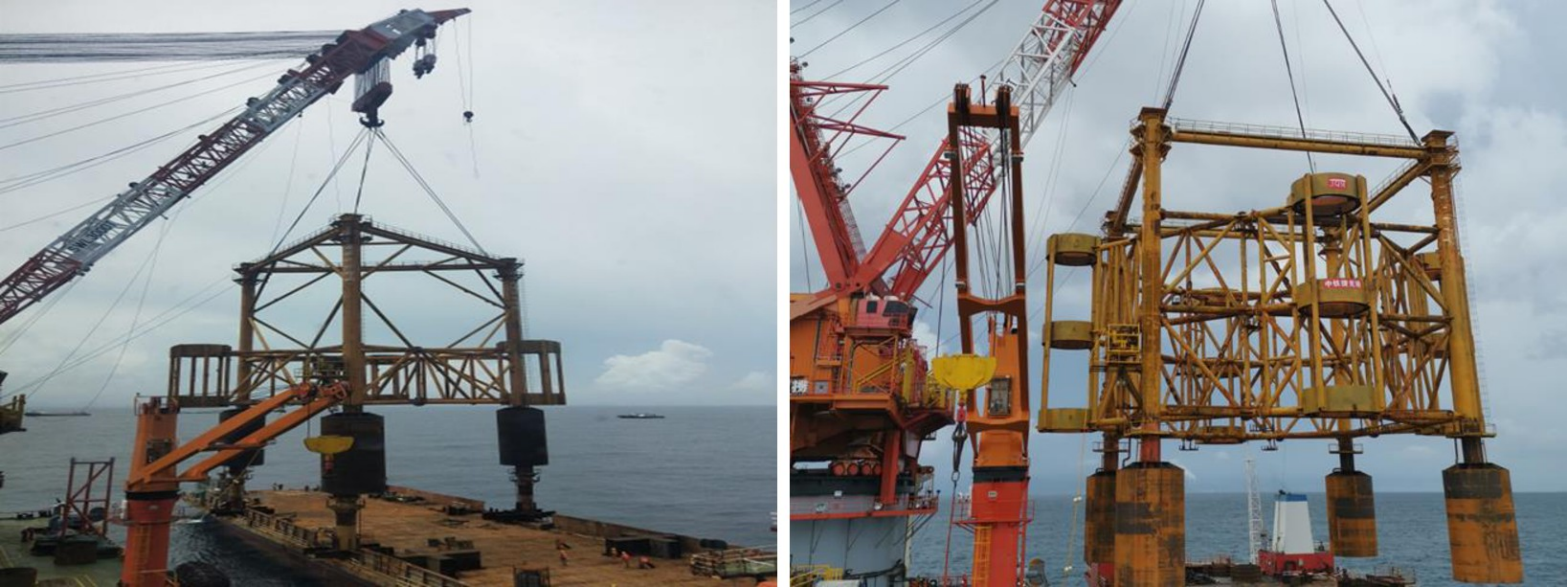
Three- and four-barrel guide frame suction drum foundation platforms.

**Fig 2:**
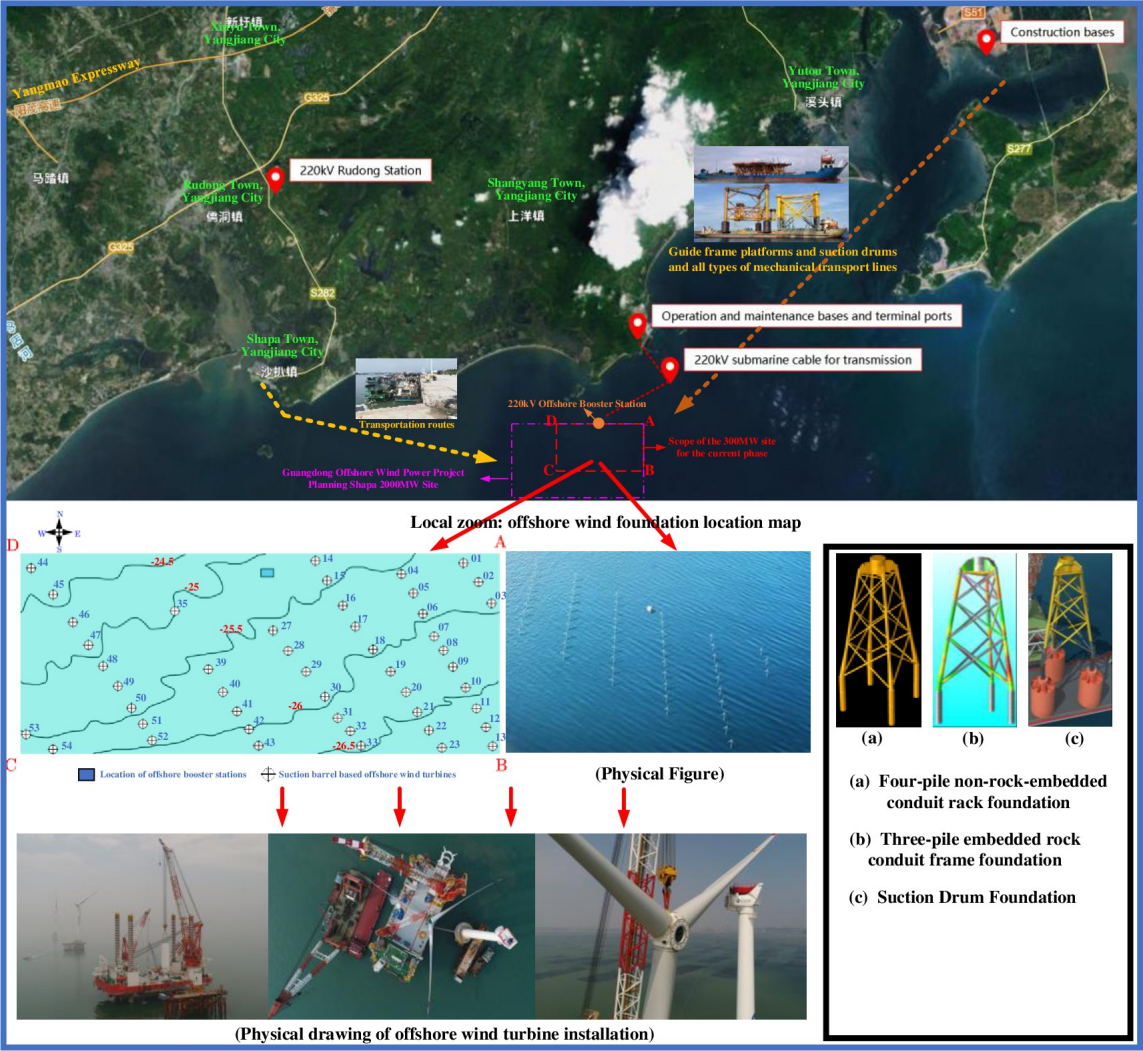
Detailed map of the geographical location of the site.

The Yangjiang Shapa Offshore Wind Power Project is situated near Shapa Town in Guangdong Province, Yangjiang City, in a sea area covering 48km2 with coordinates of A(111.665°E, 21.377°N), B(111.665°E, 21.339°N), C(111.552°E, 21.339°N) and D(111.552°E, 21.377°N). The water depth at the site ranges from 23m to 27m, and the project has a planned installed capacity of 300MW. The project’s planned installed capacity of 300MW consists of 6 wind turbines of 5.5MW and 42 wind turbines of 6.45MW, a 220kV offshore booster station, and an onshore operation and maintenance base. The wind turbines are constructed with four-pile non-rock-embedded conduit frame foundations, three-pile rock-embedded conduit frame foundations, and suction bucket foundations. Some 47 wind turbines are constructed on three-pile or four-pile guided frame suction bucket foundation platforms. For reference, please see Fig 1. The single guide frame platform is arranged with four suction drum foundations with a diameter of 5 m and a thickness of 0.03 m. The four suction drum foundations are set in a square, with a spacing of 26 m between the foundations and a length of 6.5 m. The top of the suction drum foundations is connected to the upper working platform by steel pipe columns with a diameter of 1.5 m and a thickness of 0.018 m. Details of the above are shown in Fig 2.

### 2.2. Engineering geological and hydrogeological conditions

1. Topography and geomorphology: In the project area, the change in water depth is relatively gradual. The seabed elevation ranges from -24.2m to -27.1m. The water depth gradually increases from the northwest to the southeast. The seabed topography is relatively flat, and the slope is typically less than 0.5°.
2. The stratigraphic lithology is considered. The classification of the wind farm cover is based on its origin, which includes six different layers: ① Holocene marine sedimentary layer (Q^4m^); ② Holocene marine-land transitional sedimentary layer (Q^4m+al^); ③ Quaternary residual clayey soil layer (Q^el^); ④ underlying bedrock of Mesozoic Late Cretaceous argillaceous sandstone; ⑤ sandstones; and ⑥ Palaeozoic Cambrian granitic gneiss.
3. Ground vibration parameters. The engineering geological survey report classifies the wind farm site as Type III with a peak ground vibration acceleration of 0.125g and a characteristic period of the reaction spectrum of 0.45s.
4. The site’s maximum water depth is 29 m. The maximum wind speeds for the guiding frame platform under working and non-working conditions are 20.7 m/s and 34.6 m/s, respectively. The maximum wave height of 2.5 m and a period of 6 s are employed for calculating the wave force associated with platform construction and steel sheath insertion. The platform construction and steel sheath insertion are calculated based on the water flow force of 1 m/s.

### 2.3. Analysis of the geological environment around foundation piles

**Fig 3:**
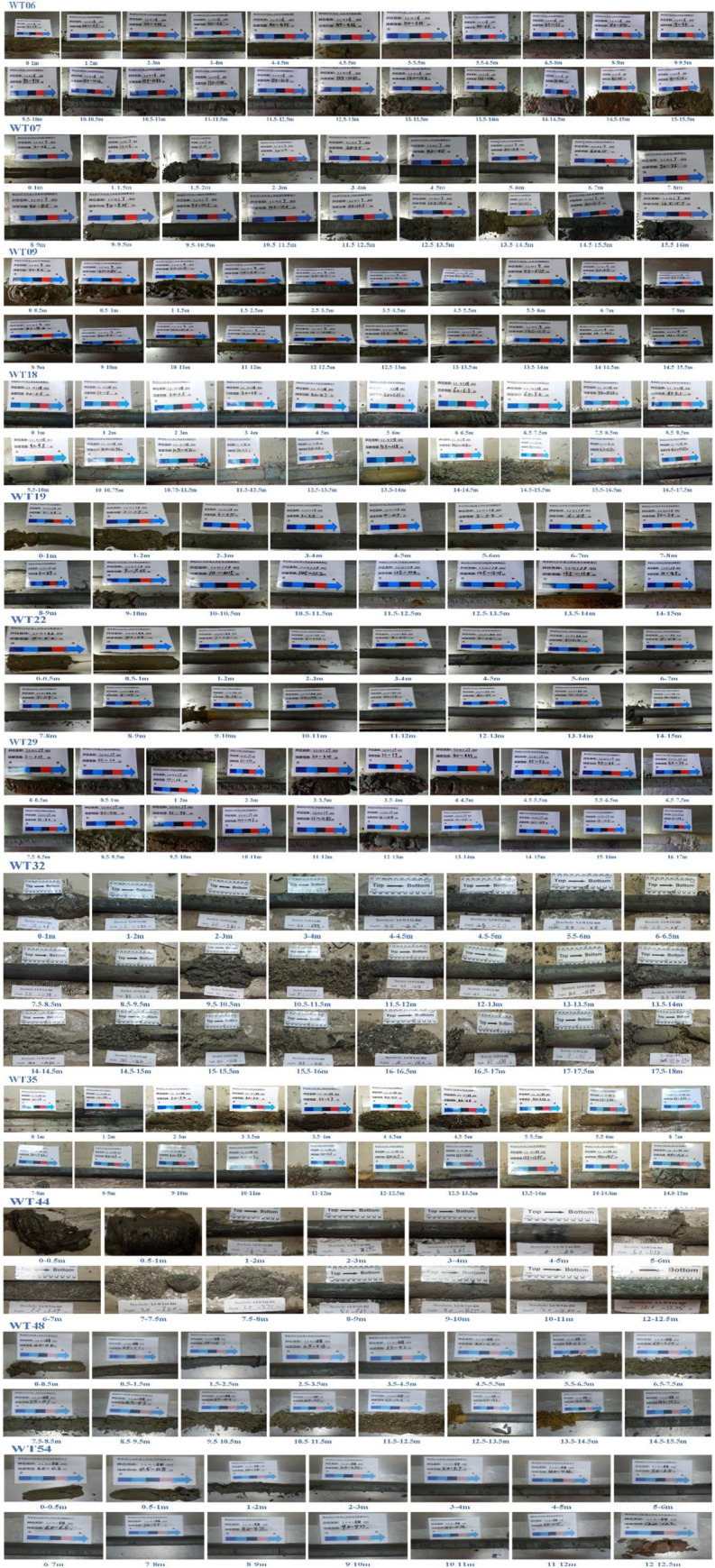
Field in-situ test.

The suction drum foundation is suitable for a wide range of soil types. Experience shows that suction buckets suit most marine structures and soil types. Suction buckets can be used to anchor a variety of floating structures in different soil conditions, including tension leg platforms. Suction buckets can be a foundation for conduit rack platforms even in complex soil conditions like dense sand and hard clay. Based on in-situ tests (refer to Fig 3) and construction records on site (Tables 1–12), three distinct soil layers have been identified within the suction drum’s depth of penetration: clay, upper clay-lower sandy soil, and upper clay-middle sandy soil-lower clay. This highlights the suction drum’s versatility in marine soils.

**Table 1:** Stratigraphy and calculation parameters for the pile location of the WT06 platform.

Soil Classification	Depth of Stratum (m)	Effective Heavy *γ*’/(kN/m^3^)	Undrained Shear Strength *S*_u_/(kPa)	Internal Friction Angle *φ*/(°)
①_1_ Silty clay	1.5	7.4	5.0	
②_2_ Powdery clay	3.8	10.3	105.0	
②_3_ Silty sandy	5	9.9		26
②_4_ Powdery clay	7.4	9.4	48	
②_4_ Sandy clay	12.5	9.5		23
②_5_ Grit	15.0	10.5		30

**Table 2:** Stratigraphy and calculation parameters for the pile location of the WT07 platform.

Soil Classification	Depth of Stratum (m)	Effective Heavy *γ*’/(kN/m^3^)	Undrained Shear Strength *S*_u_/(kPa)	Internal Friction Angle *φ*/(°)
①_1_ Silty clay	4.0	7.6	14.0	
②_4_ Silty clay	5.3	7.8	22.0	
②_4_ Silty clay	11.8	8.0	38.0	
②_5_ Coarse sand	13.3	9.2		25
②_6_ Sandy clay	14.0	10.8	110.0	
②_6_ Sandy clay	14.7	9.8	65.0	

**Table 3:** Stratigraphy and calculation parameters for the pile location of the WT09 platform.

Soil Classification	Depth of Stratum (m)	Effective Heavy *γ*’/(kN/m^3^)	Undrained Shear Strength *S*_u_/(kPa)	Internal Friction Angle *φ*/(°)
①_1_ Mud	3.5	7.4	5.0	
①_2_ Powdery clay	6.3	8.1	28.0	
②_1_ Coarse sand	8.4	9.3		23
②_3_ Grit	9.5	10.0		30
②_2_ Clay	12.0	8.9	125.0	
②_5_ Medium sand	14.1	9.5		26
②_5_ Medium sand	14.9	9.7		27

**Table 4:** Stratigraphy and calculation parameters for the pile location of the WT18 platform.

Soil Classification	Depth of Stratum (m)	Effective Heavy *γ*’/(kN/m^3^)	Undrained Shear Strength *S*_u_/(kPa)	Internal Friction Angle *φ*/(°)
①_1_ Silty clay	1.1	6.8	2.0	
①_2_ Clay	1.8	6.8	8.0	
①_2_ Clay	4.3	8.4	18	
①_2_ Clay	5.4	8.4	25	
②_1_ Medium sand	6.6	10.6		20
②_2_ Clay	8.4	7.5	35	
②_3_ Medium sand	10.5	9.0		31
②_4_ Sandy clay	11.9	10.7	35	
②_5_ Grit	16.2	10.5		27

**Table 5:** Stratigraphy and calculation parameters for the pile location of the WT19 platform.

Soil Classification	Depth of Stratum (m)	Effective Heavy *γ*’/(kN/m^3^)	Undrained Shear Strength *S*_u_/(kPa)	Internal Friction Angle *φ*/(°)
①_1_ Silty clay	2.5	7.4	7.0	
①_2_ Powdery clay	4.0	8.9	18	
②_1_ Silty sandy	6.2	9.0		25
②_1_ Medium sand	7.2	9.0		30
②_2_ Clay	9.3	8.4	50	
②_3_ Coarse sand	10.4	9.1		30
②_4_ Powdery clay	12.2	9.3	70	
②_5_ Coarse sand	13.4	9.5		28
②_5_ Coarse sand	14.4	9.5		35

**Table 6:** Stratigraphy and calculation parameters for the pile location of the WT22 platform.

Soil Classification	Depth of Stratum (m)	Effective Heavy *γ*’/(kN/m^3^)	Undrained Shear Strength *S*_u_/(kPa)	Internal Friction Angle *φ*/(°)
①_1_ Silty clay	1.9	6.8	4	
①_2_ Clay	4.2	7.5	36	
②_2_ Sandy silt	6.8	9.4		20
②_3_ Silty sandy	7.9	8.4		20
②_3_ Medium sand	9.4	9.1		25
②_4_ Clay	10.4	7.3	75	
②_4_ Clay	13.6	7.3	65	
②_6_ Coarse sand	14.6	9.8		27

**Table 7:** Stratigraphy and calculation parameters for the pile location of the WT29 platform.

Soil Classification	Depth of Stratum (m)	Effective Heavy *γ*’/(kN/m^3^)	Undrained Shear Strength *S*_u_/(kPa)	Internal Friction Angle *φ*/(°)
①_1_ Silty clay	2.9	7.5	8	
②_1_ Silty clay	5.0	9.5		20
②_2_ Silty sandy	7.1	9.5	43	
②_2_ Powdery soil	8.5	10.3	150	
②_3_ Coarse sand	10.1	9.1		35
②_4_ Clay	11.0	8.1	90	
②_5_ Medium sand	13.5	8.6		27
②_5_ Clay	14.8	8.2	89	
②_5_ Medium sand	16.0	8.5		20

**Table 8:** Stratigraphy and calculation parameters for the pile location of the WT32 platform.

Soil Classification	Depth of Stratum (m)	Effective Heavy *γ*’/(kN/m^3^)	Undrained Shear Strength *S*_u_/(kPa)	Internal Friction Angle *φ*/(°)
①_1_ Mud	0.9	4.3	4	
①_2_ Silty clay	4.1	8.1	10	
②_1_ Coarse sand	5.0	8.6		25
②_1_ Coarse sand	6.2	7.9		32
②_1_ Fine sand	8.3	9.2		33
②_1_ Grit	11.4	8.8		32
②_1_ Silty sandy	13.8	8.7		28
②_3_ Fine sand	17.8	8.9		28

**Table 9:** Stratigraphy and calculation parameters for the pile location of the WT35 platform.

Soil Classification	Depth of Stratum (m)	Effective Heavy *γ*’/(kN/m^3^)	Undrained Shear Strength *S*_u_/(kPa)	Internal Friction Angle *φ*/(°)
①_1_ Silty clay	2.0	7.0	5	
②_1_ Coarse sand	5.2	9.6		27
②_3_ Grit	7.2	10.2		32
②_4_ Clay	9.5	9.2	65	
②_5_ Fine sand	11.0	9.1		32
②_7_ Grit	12.0	10.3		38
③_2_ Coarse sand	14.8	8.9		32

**Table 10:** Stratigraphy and calculation parameters for the pile location of the WT44 platform.

Soil Classification	Depth of Stratum (m)	Effective Heavy *γ*’/(kN/m^3^)	Undrained Shear Strength *S*_u_/(kPa)	Internal Friction Angle *φ*/(°)
①_1_ Silty clay	3.5	5.5	6.0	
②_1_ Coarse sand	5.3	7.2		25
②_3_ Grit	7.0	8.8		30
②_3_ Grit	8.2	8.0		25
②_5_ Coarse sand	9.1	8.9		25
②_7_ Coarse sand	11.2	9.1		35

**Table 11:** Stratigraphy and calculation parameters for the pile location of the WT48 platform.

Soil Classification	Depth of Stratum (m)	Effective Heavy *γ*’/(kN/m^3^)	Undrained Shear Strength *S*_u_/(kPa)	Internal Friction Angle *φ*/(°)
①_1_ Silty clay	4.0	6.8	8	
②_3_ Coarse sand	7.2	9.8		35
②_3_ Coarse sand	8.6	8.5		25
②_3_ Coarse sand	12.5	9.4		33
②_4_ Clay	13.3	8.7	52	
②_5_ Grit	13.9	8.5		35
②_6_ Powdery clay	15.1	9.0	75	

**Table 12:** Stratigraphy and calculation parameters for the pile location of the WT54 platform.

Soil Classification	Depth of Stratum (m)	Effective Heavy *γ*’/(kN/m^3^)	Undrained Shear Strength *S*_u_/(kPa)	Internal Friction Angle *φ*/(°)
①_1_ Mud	0.7	6.7	3	
①_2_ Silty clay	2.6	7.0	8	
①_2_ Silty clay	5.5	7.4	18	
①_2_ Silty clay	10.4	7.4	28	
②_1_ Clay	11.7	8.2	65	

### 2.4. Recommendations for the structural foundation programme

Based on the stratigraphic conditions of the site, as determined from the investigation and similar projects, the available pile types are suction piles, steel pipe piles, bored piles, and a combination of steel pipe piles and bored piles. The pile type and length selection should be based on the requirements of the upper structure, construction conditions, and economic feasibility.

The ’Offshore Wind Farm Engineering Wind Turbine Foundation Design Code’ (NB/T10105-2018) [30] provides recommendations for selecting different foundation structures for wind turbines, as presented in Table 13.

**Table 13:** Guidelines for selecting wind turbine structures.

Water Depth /(m)	Infrastructure Type	Applicable Conditions
0~20	Pedestal Gravity Foundation / Caisson Gravity Foundation	The soil is rocky or hard with high bearing capacity, while the seabed is relatively gentle, and the areas are of low erosion.
0~20	Barrel Foundation / Suction Foundation	These are regions of sandy or soft clayey soils that have good bearing capacity and a seabed which is relatively stable.
0~30	Large Fiameter Monopile Foundations	Seabed soils with high horizontal bearing capacity in areas of low seabed scouring.
0~30	Multi-Stand Foundation	These are areas with typical geological conditions that offer more significant benefits at greater water depths.
0~30	High Pile Bearing Foundation	This refers to areas that have normal geological conditions and moderate water depths.
20~50	Conduit Racking Foundation	Areas of average geological conditions, with more pronounced advantages at deeper water depths.

Based on the analysis of Table 13 and the site survey, the suction bucket foundation is the preferred option due to its economic feasibility and safety.

## 3. Investigation of the penetration characteristics of a suction drum foundation

The safe operation of the overall structure significantly depends on whether the suction drum foundation can penetrate the design depth. Therefore, researchers mainly focus on predicting the penetration resistance of the suction penetration stage. Suction penetration exhibits varying characteristics in different geological conditions and structural dimensions due to its unique principle.

### 3.1. Analysis of penetration force in the foundation of the suction drum

**Fig 4:**
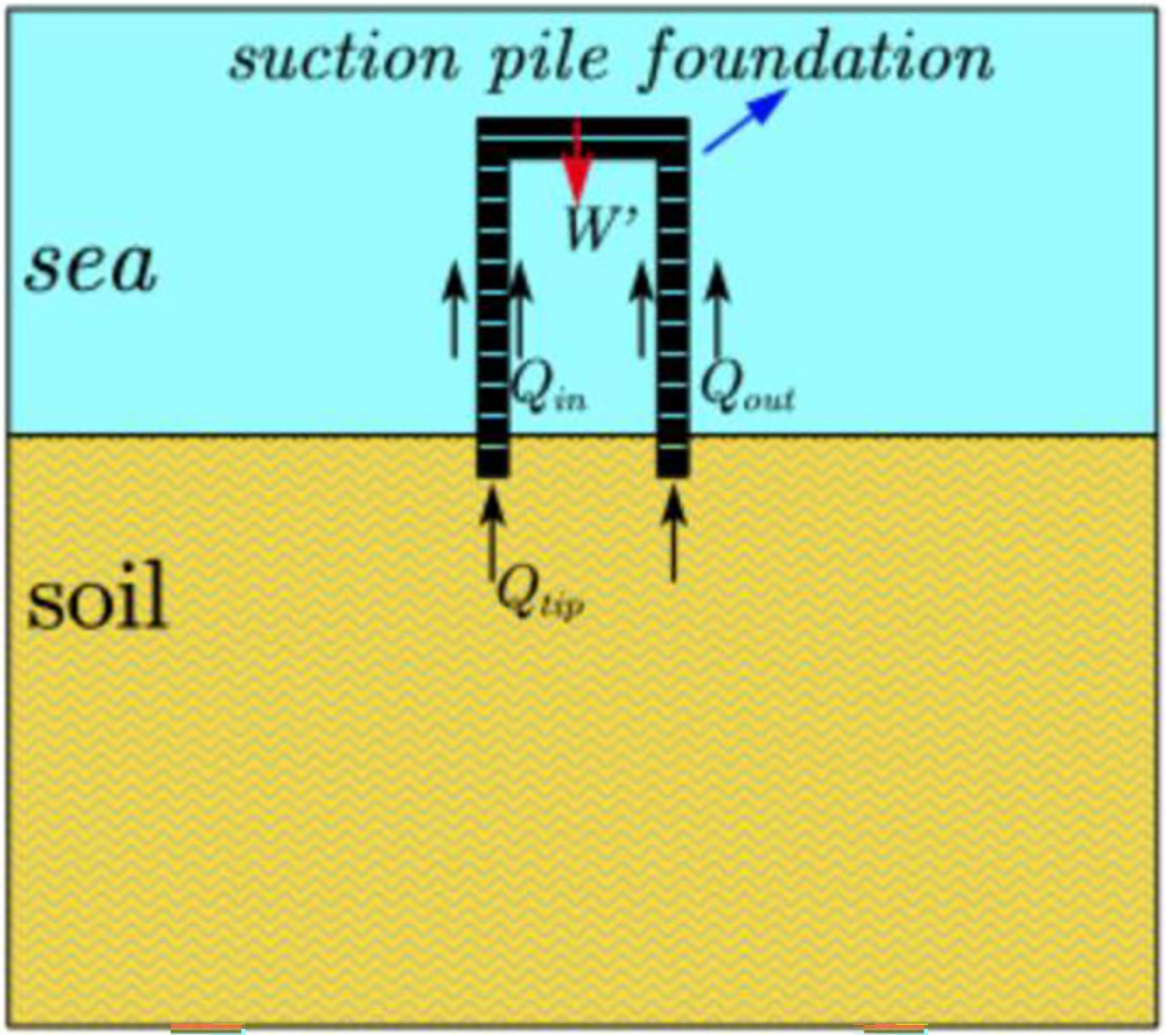
Self-weight penetration stage.

**Fig 5:**
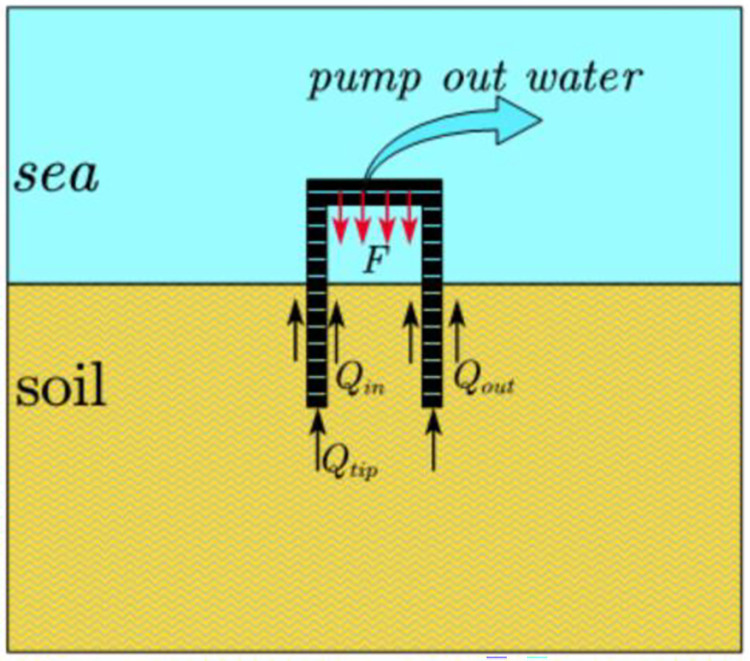
Suction penetration stage.

The suction bucket foundation penetrates the soil in two phases. Firstly, it penetrates the soil by self-weight, as shown in Fig 4. Then, it proceeds by suction, as demonstrated in Fig 5.

During the self-weight penetration phase, the foundation of the suction drum moves downwards due to its weight. The vertical equilibrium equation of the foundation can be expressed as follows:

$$Ma=W'-Q_{in}-Q_{out}-Q_{tip}$$ (1)

As shown in Eq 1: Here, *M* represents the total weight of the platform frame and suction drum foundation; *a* represents the variable acceleration of the downward movement of the suction drum foundation that varies with the change of penetration depth;*W*’ signifies the effective weight of the suction drum foundation underwater; *Q*_in_, *Q*_out_, and *Q*_tip_ represents the frictions on the inner, outer, and end sides of the suction drum foundation, respectively.

During the suction penetration stage, the structure’s weight and the soil resistance reach a balance, indicating the end of self-weight penetration. The suction drum foundation becomes sealed, and the suction of water from inside the foundation creates a pressure difference between the interior and exterior of the drum foundation, subsequently driving the suction drums further downward. This process is referred to as suction penetration. The driving force *F* is determined by multiplying the suction force value *S* with the bottom area *A*_in_ on the inner wall of the suction drum foundation. The following equation can represent the vertical balance of the suction drum foundation:

$$\begin{array}{l} Ma=W'+F-Q_{in}-Q_{out}-Q_{tip}\\ \;=W'+S•A_{\mathrm{in}}-Q_{in}-Q_{out}-Q_{tip} \end{array}$$ (2)

### 3.2. Selection of theory for calculation of penetration resistance for suction drums

Accurate derivation of the penetration resistance is crucial in determining the necessary suction force and is a critical technical aspect of construction process control.To ensure successful installation of the suction drum foundation, it is necessary to have a proper understanding and control of the penetration process. This process primarily involves the upper platform and the foundation of the drum. The key factors to consider are the weight (*W*’), negative pressure (suction) ΔU, the interaction between the negative pressure and the drum foundation area *A*_tip_, and the penetration of the total resistance to dynamic changes between the *Q*_tot_. Numerous theoretical and experimental studies have been conducted on calculating penetration resistance. In current domestic and foreign engineering design, the recommended methods are static equilibrium method and pore pressure static touch (Based-CPTU) method by API (American Petroleum Institute) [21,22] and DNV (Det Norske Veritas) [23,24]. This paper adopts the static equilibrium method to calculate penetration resistance. This paper employs the static equilibrium method for calculations. Assuming the same friction coefficients for the inner and outer sidewalls of the suction drum and disregarding the soil plug effect, the penetration depth *h*_n_ determines the penetration resistance *Q*_tot_:

$$Q_{\mathrm{tot}}=Q_{\mathrm{side}}+Q_{\mathrm{tip}}=\sum_{i=1}^{n}\pi D_{0}\Delta h_{i}\cdot (\alpha_{i\mathrm{ns}}S_{ui}+K_{i}\gamma \prime h_{i}\tan\delta_{i})+(N_{ci}S_{u\mathrm{tip}}^{AVE}+N_{qi}\gamma \prime h_{n})\cdot A_{\mathrm{tip}}$$ (3)

Eq 3 shows the following: Here are the definitions for the variables used: n represents the total number of layers; *Q*_tot_ is the total penetration resistance in kN, where *Q*_tot_ is further divided into two parts: the friction resistance *Q*_side_ along the side wall of the barrel and the end resistance *Q*_tip_ of the barrel, both in kN; *A*_tip_ is the area of the barrel end circle in m^2^. *S*_ui_ represents the undrained shear strength of the ith stratum of the soil in kPa. *N*_ci_ is the bearing capacity coefficient, whose value depends on the calculation’s purpose. It is retrieved according to the API specification [21]. The acceptable values of *N*_ci_ are presented in Table 14; *N*_qi_ is the dimensionless bearing capacity coefficient, and the acceptable range of values is presented in Table 15. $S_{\mathrm{utip}}^{AVE}$ represents the average undrained shear strength of soil at the end of the barrel in kPa. *K* is the lateral pressure coefficient, the horizontal and vertical effective positive stresses ratio. It is acceptable to use the measured value of *K*. In case there is no measured value, for cohesive soils, *K* = 0, and for cohesionless soils, *K* = 0.8. Alternatively, *K* can be calculated using the formula *K* = (1-sin*φ*), where *φ* represents the soil layer’s friction angle. *γ*’ represents the effective gravity of the soil in kN/m^3^, while *δ* is the friction angle between the barrel and the soil in °. It is acceptable to retrieve *δ* according to the literature [21] if no measured value exists. On the other hand, *α*_ins_ represents the viscosity coefficient of the soil, which is equal to the inverse of the sensitivity of the soil (α_ins_ = 1/*S*_t_). *α*_ins_ is generally expressed as a range of values between 0.2 and 0.5. Expressing α_ins_ within the range of 0.2 to 0.5 is acceptable, with a maximum value of 1.0. The specific value is calculated using Eq 4:

$$a_{ins}=\{\begin{array}{ll} \;1 & \psi \leq 0.25;\\ 0.5\psi^{-0.5} & 0.25<\psi \leq 1.0\\ 0.5\psi^{-0.25} & \psi >1.0 \end{array}$$ (4)

**Table 14:** Recommended *N*_ci_ Coefficients.

Purpose of the solution	surface shape	*N*_c_	Notification Bulletin
Solving for pile end resistance	square-shaped body	7.5	
Solving for the ultimate vacuum that causes soil plug failure	orbicular	6.2~9.0	Based on pile penetration rate
Solving for the sinking resistance of a protrusion	variant	5~13.5	

**Table 15:** *N*_γ_, *N*_q_, *N*_c_ coefficients.

*φ*(°)	*N*_c_	*N*_q_	*N*_γ(H)_	*N*_γ(v)_	*φ* (°)	*N*_c_	*N*_q_	*N*_γ(H)_	*N*_γ(v)_
0	5.14	1.00	0	0	24	19.33	9.61	6.90	9.44
2	5.69	1.20	0.01	0.15	26	22.25	11.83	9.53	12.54
4	6.17	1.43	0.05	0.34	28	25.80	14.71	13.13	16.72
6	6.82	1.72	0.14	0.57	30	30.15	18.40	18.09	22.40
8	7.52	2.06	0.27	0.86	32	35.50	23.18	24.95	30.22
10	8.53	2.47	0.47	1.22	34	42.18	29.45	34.54	41.06
12	9.29	2.97	0.76	1.69	36	50.61	37.77	48.08	56.31
14	10.37	3.58	1.16	2.29	38	61.36	48.92	67.43	78.03
16	11.62	4.33	1.72	3.06	40	75.36	64.23	95.51	109.11
18	13.09	5.25	2.49	4.07	42	93.69	85.36	136.72	155.55
20	14.83	6.40	3.54	5.39	44	118.41	115.35	198.77	224.64
22	16.89	7.82	4.96	7.13	45	133.86	134.86	240.95	271.76

Eq 4 specifies that the value of constraint *a*_ins_ cannot exceed 1. *ψ* is defined as the ratio of *S*_*u*_ and $P_{O}^{'}$, where $P_{O}^{'}$ represents the effective vertical stress at the calculation point.

### 3.3. Limiting negative pressure value for the suction drum base penetration

Under negative pressure, the foundation of the suction drum penetrates, and the upper part of the drum generates pressure due to the suction force, forming a downward force that drives the foundation to sink to the design depth. During this stage, it is crucial to ensure that the suction force required is sufficient to overcome the penetration resistance and allow the foundation to sink smoothly. It is also necessary to prevent excessive suction force that could cause soil upwelling in the barrel, leading to soil plugs and the foundation failing to reach the design depth. Infiltration damages such as pipe surges and sand flow must also be prevented in sandy soils.

Determining the critical suction value (ΔU_cirt_), also known as the "ultimate suction value," during the suction sinking process is crucial to avoid the abovementioned issues. If ΔU_cirt_ value is not determined correctly, it can cause an overall reverse failure of the end of the suction drum foundation, leading to a large influx of soil into the drum foundation and creating a vacuum pressure. Eqs 5 and 6 are used to calculate the ΔU_cirt_ value:

In soils with high clay content:

$$\Delta U_{crit}=N_{c}\cdot S_{utip}^{AVE}+\frac{A_{inside}\cdot {\left(\alpha_{ins}\cdot S_{uDSS}\right)}_{AVE}}{A_{in}}$$ (5)

In soils that lack cohesion:

$$\Delta U_{crit}=\{\pi -\arctan[5{\left(L/D\right)}^{0.85}]\frac{2(\pi -1)}{\pi }\}L\gamma '$$ (6)

Eqs 5 and 6 are provided as follows: Here, *A*_*inside*_ represents the cross-sectional area of the barrel wall in square meters, *S*_*uDSS*_ represents the viscous soil straight shear strength in kilopascals, *L* represents the suction barrel height in meters, and *D* represents the suction barrel diameter in meters. The rest of the symbols carry the same meanings provided by Eq 3.

We define the maximum suction force applied to the suction drum foundation as the allowable suction force (ΔU_allow_), also called the "upper limit of safe suction value." Eq 7 is used to calculate the value of ΔU_allow_ as follows:

$$\Delta U_{\mathrm{allow}}=\frac{\Delta U_{\mathrm{crit}}}{k}$$ (7)

The Eq 8 calculates the maximum suction available in practical engineering.


$$\Delta U_{\mathrm{allow}}=\min(\frac{\Delta U_{\mathrm{crit}}}{k},\;\gamma_{w}h)$$ (8)

Eqs 7 and 8describe the variables involved in the calculation as follows: *k* represents the safety factor, which is generally not less than 1.25 and has a recommended value of 1.5; *γ*_w_ is the seawater capacity measured in kN/m^3^; and *h* represents the vertical distance from sea level to the top of the suction drum foundation, measured in meters.

The suction force required by the foundation of the suction drum, also known as the pressure required to penetrate the specified depth, is denoted by ΔU_req_. Formula (9) is used to calculate the value of ΔU_req_.


$$\Delta U_{\mathrm{req}}=\frac{Q_{\mathrm{tot}}-W'}{A_{\mathrm{in}}}$$ (9)

Eq 9, Here, *W*’ refers to the combined weight of the suction drum foundation and the weight distributed by the upper platform in kiloNewtons, At the same time, *A*_in_ represents the effective cross-sectional area of the suction drum foundation in square meters.

### 3.4. Numerical simulation technique for suction drum foundation penetration

**Fig 6:**
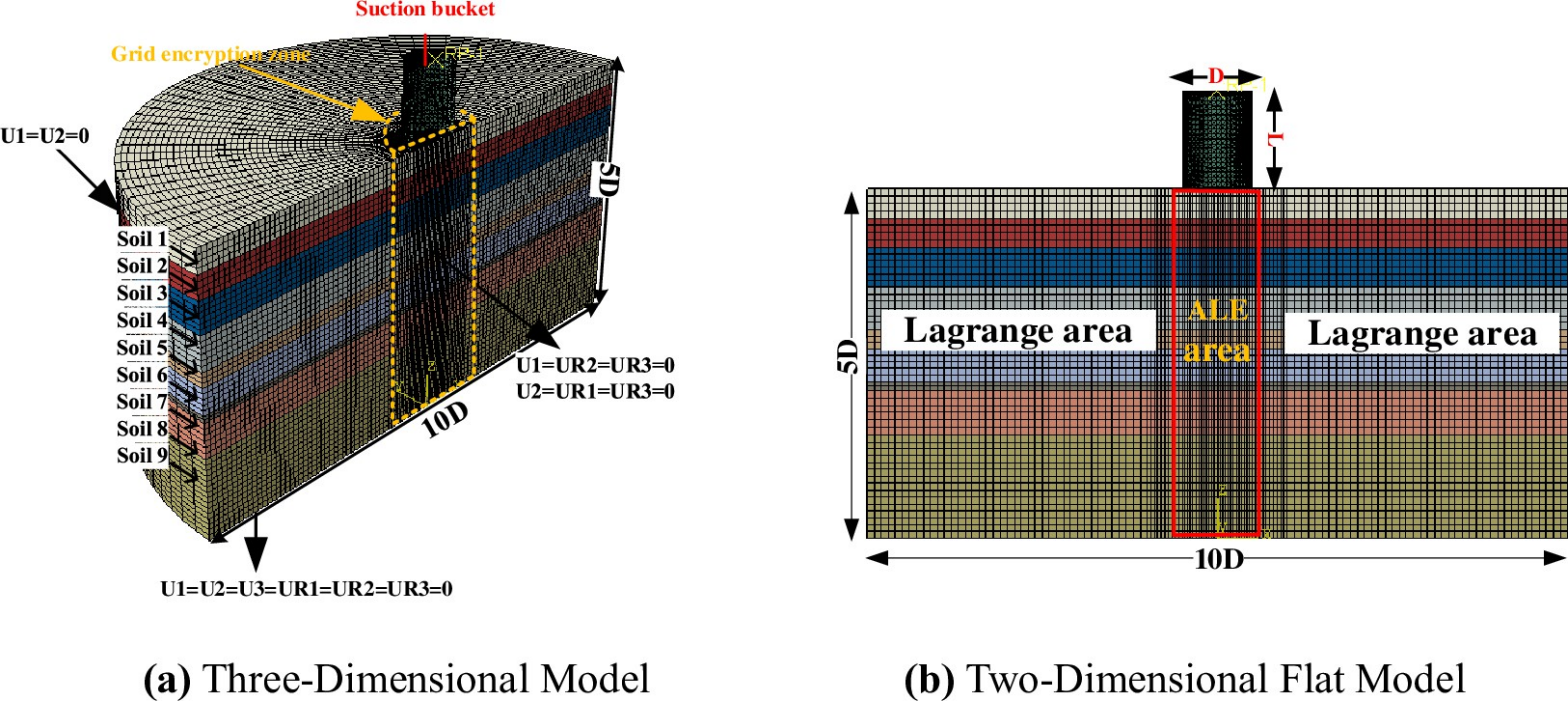
Basic model drawing of suction bucket.

(1) Modelling method. This paper employs the ALE (Arbitrary Lagrangian-Eulerian) method in the numerical software ABAQUS/Explicit to simulate and analyze the suction drum foundation penetration process. The ALE method offers advantages from pure Lagrangian and Eulerian methods while avoiding mesh distortion issues from the Lagrangian method in conventional finite elements. It is a suitable choice for computational analysis of pile-soil penetration processes.

(2) Geometry and mesh. Geometry and mesh. We created a 1/2 model of the suction drum foundation to speed up the solution speed due to its structural symmetry. We utilized the ALE (Arbitrary Lagrangian-Eulerian) method in ABAQUS to simulate its penetration. Fig 6 illustrates the original model, which had a barrel length of L = 7m, barrel diameter of D = 5m, and barrel wall thickness of t = 0.03m. We used typical soil model proportions of 10D for length and 5D for width and height to preclude boundary effects. The friction coefficient (*μ*) for contact between the pile and soil typically ranges from 0.1 to 0.42 [31–33]. To fully constrain the bottom of the soil body, set U1 = U2 = U3 = UR1 = UR2 = UR3 = 0 in the x, y, and z directions. For the sides of the soil body, apply constraints in the x and y directions by setting U1 = U2 = 0, U1 = UR2 = UR3 = 0, U2 = UR1 = UR3 = 0. The soil body’s surface is a free boundary with no constraints.

Tetrahedral cell C3D4H is implemented as the mesh type for the barrel, whereas linear reduced integral cell C3D8R is chosen for the surrounding soil. Employing the Mohr-Coulomb elastoplastic model allows for a better reflection of the nonlinearity of the soil structure, with all necessary parameters obtained from geotechnical tests. We do not factor in the impact of strain softening on the undrained shear strength in the simulation.

The mesh delineation method follows a specific approach that encrypts the pile-soil contact surface area mesh. It is recommended to use an encrypted mesh size of 0.05D and an encrypted mesh range of 2D [34]. Additionally, the mesh size should increase gradually from the center to the outward direction.

**Fig 7:**
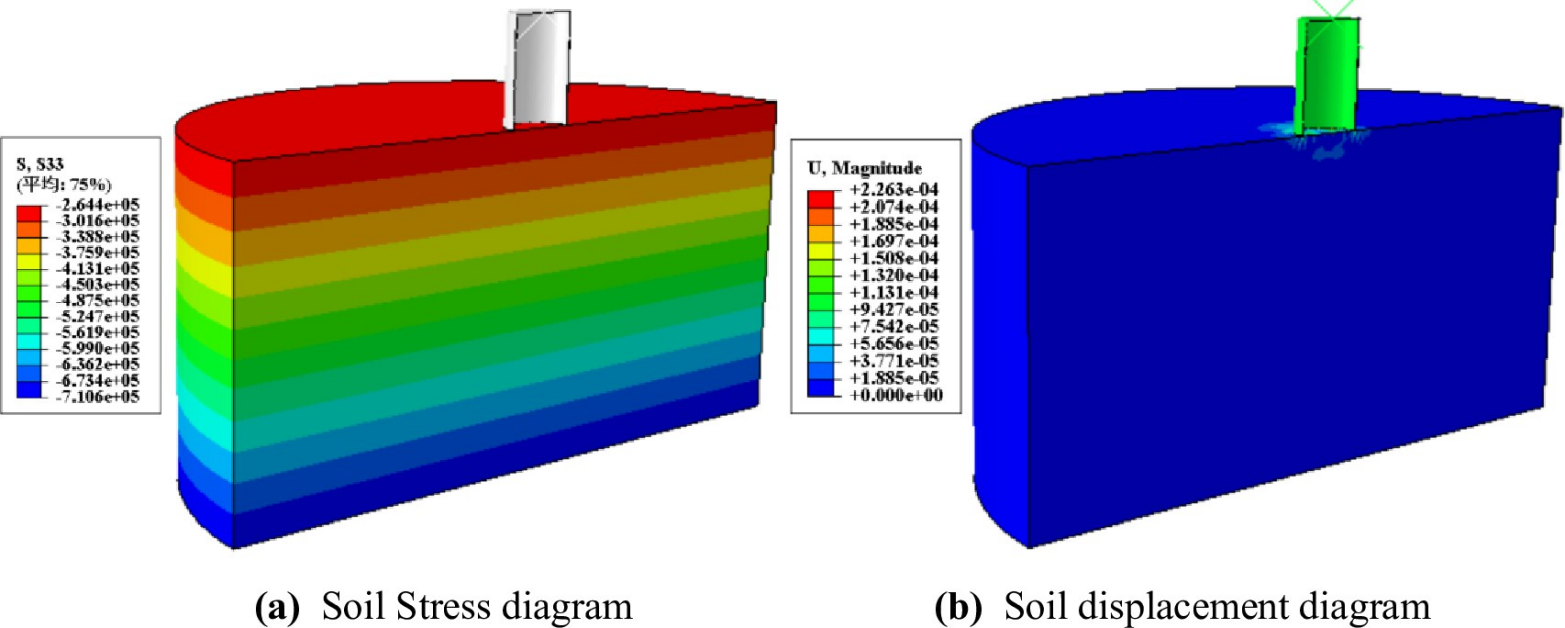
Geostress balance diagram.

**Fig 8:**
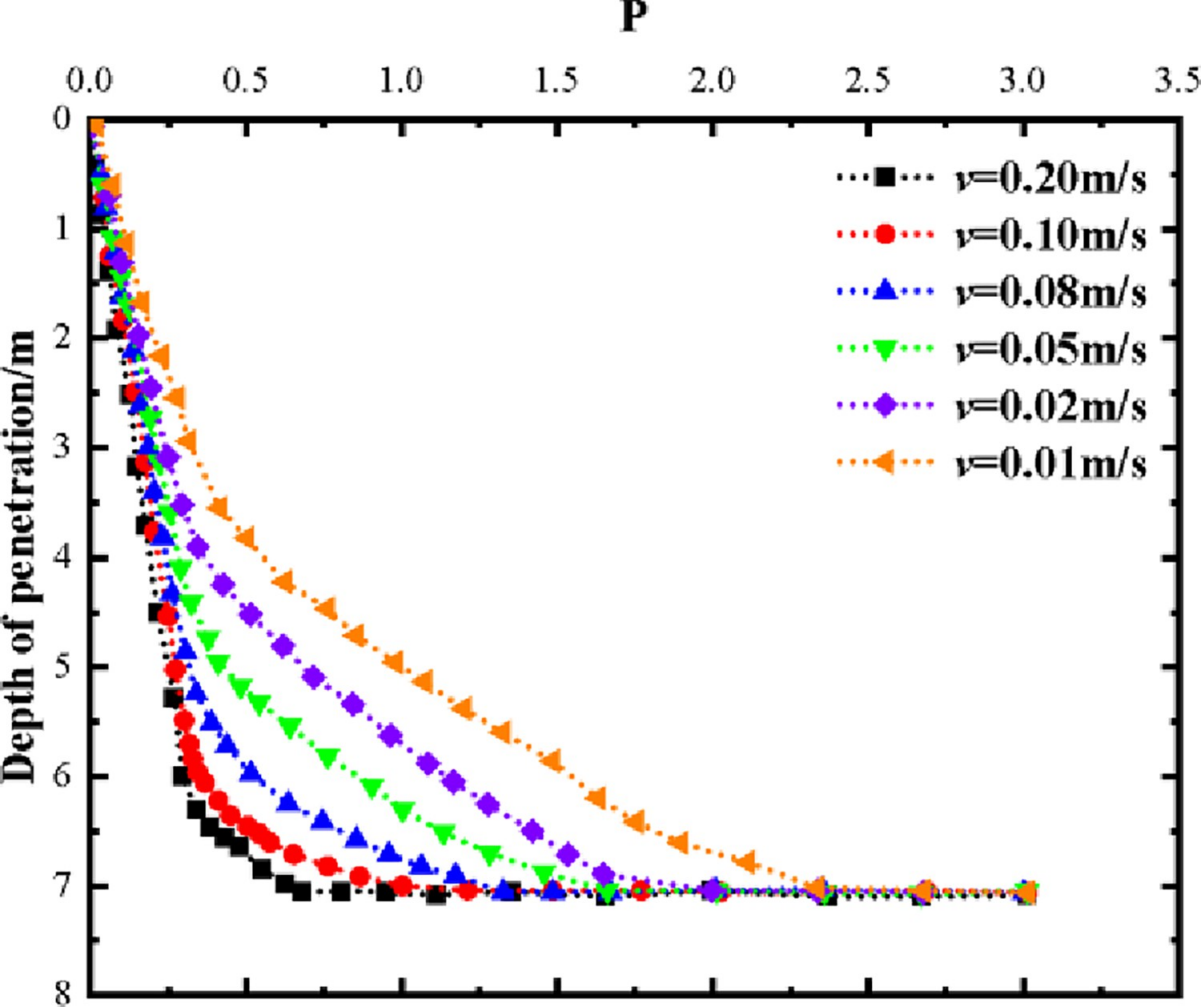
Effect of P value on depth of penetration.

(3) Geostress balance. The ALE method imports the output database (ODB) file to calculate the initial geo-stress and displacement of the soil via the static general analysis step. It then forms the ODB file and cancels the initial displacement by applying the geo-stress balance analysis step. The stress and initial displacement fields achieve geo-stress equilibrium, generating displacement magnitudes below 10^-4^m. The geo-stress field exhibits uniform distribution in a layer shape and steadily increases with depth; this effect aligns with the actual project. Fig 7 displays a visual representation.

(4) Convergence analysis of mesh and sinking velocity. For the purpose of ensuring adequate computational accuracy, the grid size of the encrypted area was examined using the suction drum foundation with a diameter of D = 5m, drum wall thickness of t = 0.03m, and drum length of L = 7m as an example. The grid size d to drum thickness t ratio was denoted as P. The effect of P on penetration depth at varying velocities is displayed in Fig 8.

As demonstrated in Fig 8, the suction drum foundation can penetrate to a predetermined depth of 7 meters at varying velocities when P is greater than or equal to 2.5. At the same time, for P less than or equal to 2.25, the penetration depth reduces with decreasing velocity at the same grid size. When the mesh size is too small, or the penetration speed is too low, the suction drum’s foundation penetration amplifies the mesh deformation process’s singularity. This results in the computation failing to converge. To ensure penetration of the suction drum foundation to a predetermined depth, different mesh sizes should be used for different velocities, and different velocities should be used for different mesh sizes. The appropriate grid size for the encrypted area can be selected from the literature [34]. The P value of the selection is 8.3, which is higher than the critical value of 2.5. The suction drum foundation can penetrate to a predetermined depth of 7m at various speeds, fulfilling the requirements above.

**Fig 9:**
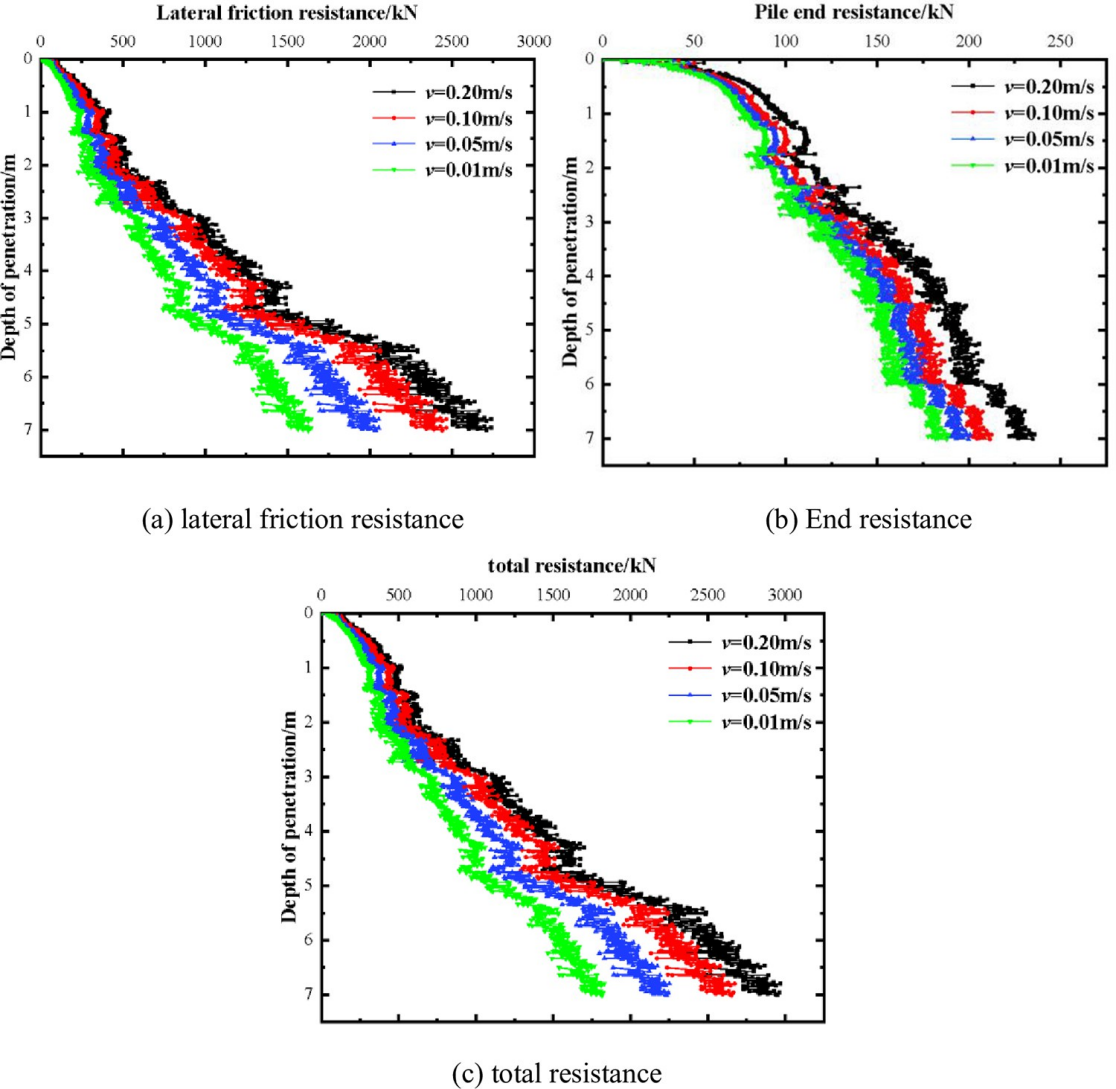
Examination of the effect of sinking velocity on penetration resistance.

The choice of penetration rate for the suction drum foundation construction varies, and it significantly impacts the stability of the drum penetration in practical engineering—the study model employed speeds of 0.01, 0.05, 0.1, and 0.2 m/s. Fig 9 illustrates the variation between the side friction resistance, end resistance, and total resistance of the suction bucket for barrel foundation across different sinking rates. Each penetration resistance of the barrel foundation sinking converges as the sinking rate diminishes progressively from 0.2 m/s to 0.01 m/s.

The computation time of each model under identical working conditions is proportionate to the sinking duration of the suction drum foundation, owing to the computational features of the explicit finite element method. This implies an inverse proportion between the computation time and the sinking speed of said foundation, indicating that as the sinking rate increases, the computation time diminishes. Considering the influence of sinking speed and calculation efficiency, this paper finally adopts the sinking speed of 0.1m/s to simulate the penetration process of the suction drum foundation.

Based on the current construction, Table 16 displays the dimensions of the finite element model for the suction drum foundation. Table 17 provides the material parameters for the drum foundation, whereas Tables 1 to 12 present the detailed soil material calculation parameters. The soil’s shear expansibility is ignored during the sinking and penetration of suction drum foundations due to the Mohr-Coulomb elastic-plastic model inherent in the ABAQUS software. This model prohibits the shear expansibility angle from zero; the minimum value of 0.1 is used instead.

**Table 16:** Dimensions and weights of suction drum foundations for various pile foundation positions.

Base position	Foundation length L/(m)	Foundation Diameter D/(m)	Barrel wall thickness t/(m)	Weights (kN)
WT06	7.0	5.0	0.03	430.1
WT07	10.5	5.0	0.03	556.9
WT09	9.0	5.0	0.03	502.5
WT18	9.0	5.0	0.03	502.5
WT19	8.0	5.0	0.03	466.3
WT22	8.0	5.0	0.03	466.3
WT29	7.0	5.0	0.03	430.1
WT32	7.0	5.0	0.03	430.1
WT35	8.0	5.0	0.03	466.3
WT44	7.0	5.0	0.03	430.1
WT48	6.3	5.0	0.03	404.7
WT54	9.0	5.0	0.03	502.5

**Table 17:** Parameters of the suction bucket.

Parameters	Densities *ρ*/(kg•m^-3^)	Elasticity Modulus *E*/(GPa)	Poisson’s Ratio (*v*)
Bucket	7800	210	0.3

## 4. Comparing measured data with theoretical calculations and numerical simulations

### 4.1. Analysis of the feasibility of penetration

**Fig 10:**
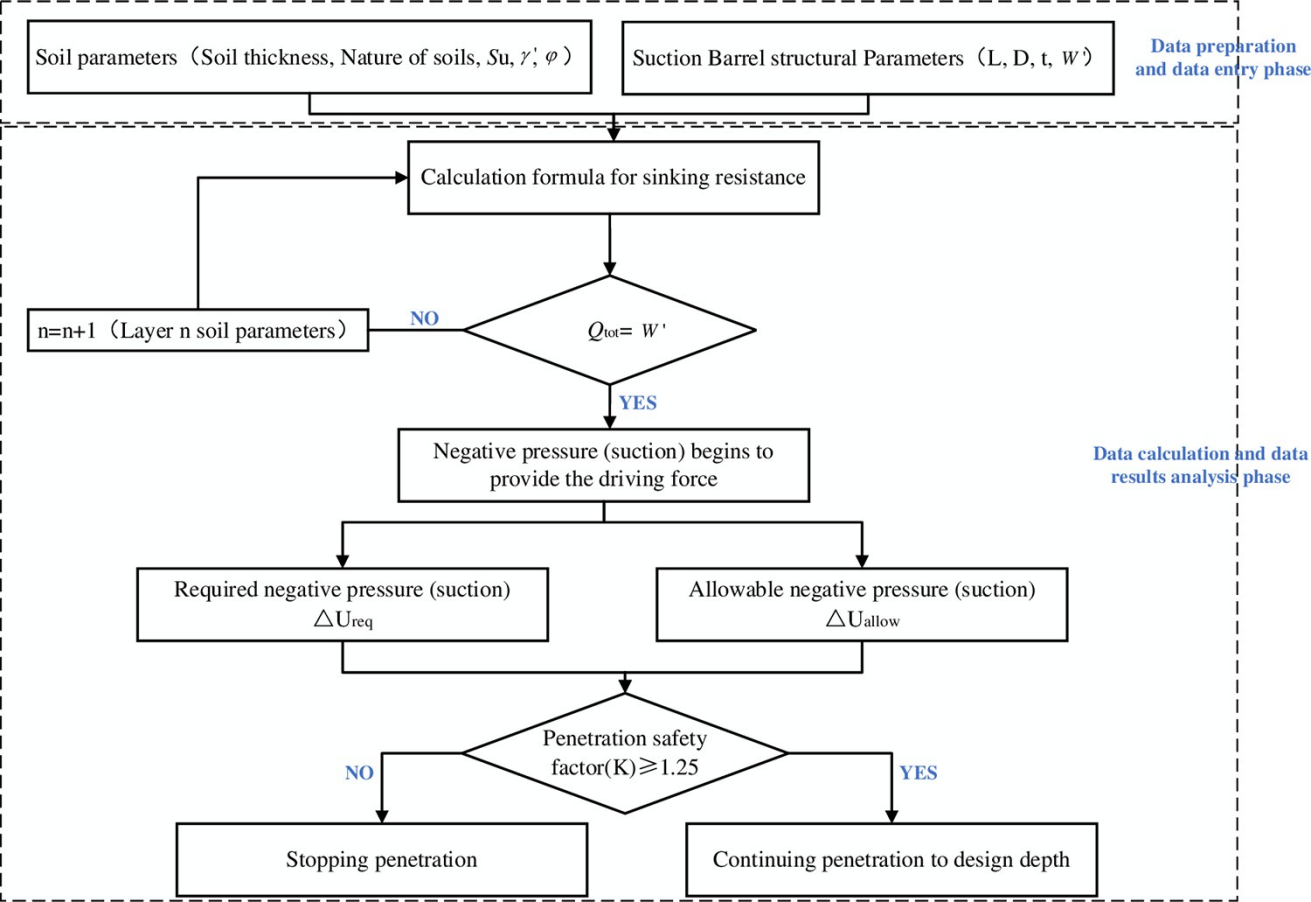
Feasibility process for suction drum sinking.

Conducting a penetration feasibility analysis prior to construction is crucial to identifying potential safety risks in advance. The penetrability of each soil layer is evaluated based on the safety coefficient calculation, and soil layers with a safety coefficient (K)<1.25 are treated to ensure the smooth penetration of the suction drum foundation to the intended depth. When conducting a penetration feasibility analysis, note that dividing the soil into smaller layers leads to greater calculation accuracy and increases the corresponding workload. Based on engineering experience, soil layers are typically 0.2~1.0m thick. However, in the suction drum foundation penetration analysis case, a layering thickness of 0.5m is deemed suitable. Fig 10 shows the suction drum foundation penetration feasibility analysis process.

The safety coefficient K is used to estimate the feasibility and penetration depth of the suction drum foundation at twelve different machine positions (WT06, WT07, WT09, WT18, WT19, WT22, WT29, WT32, WT35, WT44, WT48, and WT54) in the construction project. When K is greater than or equal to 1.25 [35], it indicates that the foundation can be safely penetrated.

By analyzing and calculating, the sinking feasibility analysis data for the suction drum foundations of the twelve machine positions mentioned earlier (WT06, WT07, WT09, WT18, WT19, WT22, WT29, WT32, WT35, WT44, WT48, and WT54) are presented in Tables 18–29.

**Table 18:** Data from the feasibility analysis of WT06 machine penetration.

Open suction penetration depth to penetration design depth/(m)	Theoretical ΔU_allow_ /(kPa)	Measured ΔU_allow_ /(kPa)	Theoretical ΔU_req_ /(kPa)	Measured ΔU_req_ /(kPa)	Theoretical K	Measured K
3.2	285.18	246.93	0.00	0.00	2.16	3.06
3.5	288.12	249.90	21.43	18.44	2.00	2.64
4.0	293.02	184.97	20.83	45.27	2.05	1.83
4.5	297.92	206.23	32.03	64.48	1.93	1.76
5.0	302.82	228.23	44.40	85.38	1.82	1.69
5.5	307.72	269.50	60.63	92.00	1.68	1.83
6.0	312.62	274.40	81.09	115.02	1.54	1.67
6.5	317.52	279.3	102.32	137.89	1.41	1.54
7.0	322.42	284.20	124.32	161.72	1.31	1.43

**Table 19:** Data from the feasibility analysis of WT07 machine penetration.

Open suction penetration depth to penetration design depth/(m)	Theoretical ΔU_allow_ /(kPa)	Measured ΔU_allow_ /(kPa)	Theoretical ΔU_req_ /(kPa)	Measured ΔU_req_ /(kPa)	Theoretical K	Measured K
6.4	316.54	244.04	0.00	0.00	2.60	2.91
6.5	317.52	245.00	0.00	3.58	2.49	2.83
7.0	322.42	249.90	16.90	22.37	2.21	2.51
7.5	327.32	254.80	35.85	42.32	1.99	2.25
8.0	332.22	259.70	55.40	62.92	1.80	2.03
8.5	337.12	264.60	75.56	84.17	1.65	1.85
9.0	342.02	269.50	96.33	106.04	1.52	1.70
9.5	346.92	274.40	117.70	128.51	1.41	1.57
10.0	351.82	279.30	139.38	151.59	1.31	1.46
10.5	362.72	284.20	161.98	175.01	1.25	1.36

**Table 20:** Data from the feasibility analysis of WT09 machine penetration.

Open suction penetration depth to penetration design depth/(m)	Theoretical ΔU_allow_ /(kPa)	Measured ΔU_allow_ /(kPa)	Theoretical ΔU_req_ /(kPa)	Measured ΔU_req_ /(kPa)	Theoretical K	Measured K
6.53	264.46	207.74	0.00	0.00	2.38	2.65
7.0	268.44	226.62	0.00	16.48	2.11	2.48
7.5	274.72	247.13	17.62	35.04	1.91	2.32
8.0	281.32	268.19	35.38	54.73	1.74	2.18
8.5	288.27	279.30	71.83	115.67	1.46	1.68
9.0	295.55	284.20	94.42	146.58	1.34	1.51

**Table 21:** Data from the feasibility analysis of WT18 machine penetration.

Open suction penetration depth to penetration design depth/(m)	Theoretical ΔU_allow_ /(kPa)	Measured ΔU_allow_ /(kPa)	Theoretical ΔU_req_ /(kPa)	Measured ΔU_req_ /(kPa)	Theoretical K	Measured K
6.68	254.14	230.69	0.00	0.00	2.08	2.83
7.0	320.46	264.60	12.06	6.69	2.32	2.95
7.5	325.36	269.50	29.79	26.48	2.09	2.60
8.0	330.26	274.40	48.08	45.98	1.90	2.33
8.5	335.16	279.30	93.02	128.02	1.53	1.60
9.0	340.83	284.20	113.59	159.36	1.42	1.44

**Table 22:** Data from the feasibility analysis of WT19 machine penetration.

Open suction penetration depth to penetration design depth/(m)	Theoretical ΔU_allow_ /(kPa)	Measured ΔU_allow_ /(kPa)	Theoretical ΔU_req_ /(kPa)	Measured ΔU_req_ /(kPa)	Theoretical K	Measured K
6.05	209.40	199.47	0.00	0.00	2.13	2.63
6.5	210.83	219.19	0.00	42.82	1.72	2.06
7.0	213.49	242.59	14.42	68.64	1.54	1.90
7.5	330.26	279.30	20.94	47.31	2.28	2.35
8.0	335.16	284.20	43.84	71.60	1.99	2.09

**Table 23:** Data from the feasibility analysis of WT22 machine penetration.

Open suction penetration depth to penetration design depth/(m)	Theoretical ΔU_allow_ /(kPa)	Measured ΔU_allow_ /(kPa)	Theoretical ΔU_req_ /(kPa)	Measured ΔU_req_ /(kPa)	Theoretical K	Measured K
6.19	192.41	195.87	0.00	0.00	1.62	2.58
6.5	195.35	207.12	8.82	8.46	1.47	2.50
7.0	206.33	225.83	22.66	22.72	1.41	2.38
7.5	230.30	244.97	37.49	37.81	1.42	2.28
8.0	281.26	264.98	61.40	66.57	1.52	2.04

**Table 24:** Data from the feasibility analysis of WT29 machine penetration.

Open suction penetration depth to penetration design depth/(m)	Theoretical ΔU_allow_ /(kPa)	Measured ΔU_allow_ /(kPa)	Theoretical ΔU_req_ /(kPa)	Measured ΔU_req_ /(kPa)	Theoretical K	Measured K
6.54	316.54	279.69	0.00	0.00	2.51	3.29
7.0	321.44	284.20	25.01	18.80	2.18	2.88

**Table 25:** Data from the feasibility analysis of WT32 machine penetration.

Open suction penetration depth to penetration design depth/(m)	Theoretical ΔU_allow_ /(kPa)	Measured ΔU_allow_ /(kPa)	Theoretical ΔU_req_ /(kPa)	Measured ΔU_req_ /(kPa)	Theoretical K	Measured K
5.66	193.51	167.24	0.00	0.00	2.15	2.38
6.0	193.76	181.25	0.00	15.06	1.89	2.21
6.5	196.14	202.84	0.00	42.77	1.60	1.97
7.0	199.77	225.52	17.64	69.45	1.43	1.81

**Table 26:** Data from the feasibility analysis of WT35 machine penetration.

Open suction penetration depth to penetration design depth/(m)	Theoretical ΔU_allow_ /(kPa)	Measured ΔU_allow_ /(kPa)	Theoretical ΔU_req_ /(kPa)	Measured ΔU_req_ /(kPa)	Theoretical K	Measured K
5.41	149.84	173.83	0.00	0.00	1.60	2.43
5.5	150.76	177.92	0.00	7.02	1.58	2.30
6.0	156.24	201.48	0.00	32.07	1.40	2.09
6.5	162.49	225.94	5.52	58.98	1.25	1.91
7.0	187.52	251.33	24.39	87.80	1.26	1.77
7.5	319.48	279.30	29.07	68.34	2.08	2.10
8.0	324.38	284.20	56.22	97.02	1.80	1.85

**Table 27:** Data from the feasibility analysis of WT44 machine penetration.

Open suction penetration depth to penetration design depth/(m)	Theoretical ΔU_allow_ /(kPa)	Measured ΔU_allow_ /(kPa)	Theoretical ΔU_req_ /(kPa)	Measured ΔU_req_ /(kPa)	Theoretical K	Measured K
6.54	167.91	160.71	0.00	0.00	1.73	2.32
7.0	172.75	177.21	0.00	17.46	1.53	2.14

**Table 28:** Data from the feasibility analysis of WT48 machine penetration.

Open suction penetration depth to penetration design depth/(m)	Theoretical ΔU_allow_ /(kPa)	Measured ΔU_allow_ /(kPa)	Theoretical ΔU_req_ /(kPa)	Measured ΔU_req_ /(kPa)	Theoretical K	Measured K
5.34	184.19	154.45	0.00	0.00	2.22	2.28
5.5	184.97	161.01	0.00	7.01	1.87	2.20
6.0	187.12	182.58	0.00	30.97	1.61	2.00
6.3	188.52	195.95	0.00	49.56	1.92	1.86

**Table 29:** Data from the feasibility analysis of WT54 machine penetration.

Open suction penetration depth to penetration design depth/(m)	Theoretical ΔU_allow_ /(kPa)	Measured ΔU_allow_ /(kPa)	Theoretical ΔU_req_ /(kPa)	Measured ΔU_req_ /(kPa)	Theoretical K	Measured K
7.1	309.04	250.88	0.00	0.00	2.58	2.96
7.5	316.76	254.80	9.95	13.06	2.33	2.71
8.0	324.72	259.70	25.94	30.43	2.14	2.45
8.5	333.00	264.60	42.61	48.15	1.97	2.23
9.0	340.06	269.50	59.72	66.21	1.83	2.05

The table above indicates that the self-weight penetration depth of the WT48 suction drum base with a drum length of 6.3m is 5.34m. The depth of self-weight penetration in suction drum foundations for WT06, WT29, WT32, and WT44, with a drum length of 7.0m, ranged from 3.2m to 6.54m. With an 8.0m long drum, the depths at which the suction drum foundations of WT19, WT22, and WT35 penetrated the ground due to their weight ranged from 5.41m to 6.19m. The self-weight penetration depths of the suction drum foundations, namely WT09, WT18, and WT54, with drum lengths of 9.0m, ranged from 6.53m to 7.10m. Likewise, the WT07 suction drum foundation with a drum length of 10.5m had a self-weight penetration depth of 6.40m. The table above shows that the measured K value is larger than the theoretical K, indicating a high level of safety in construction. Both K values are ≥1.25. If the K value is <1.25, cyclic pressurization techniques can penetrate the suction drum’s foundation until the design depth is reached.

**Fig 11:**
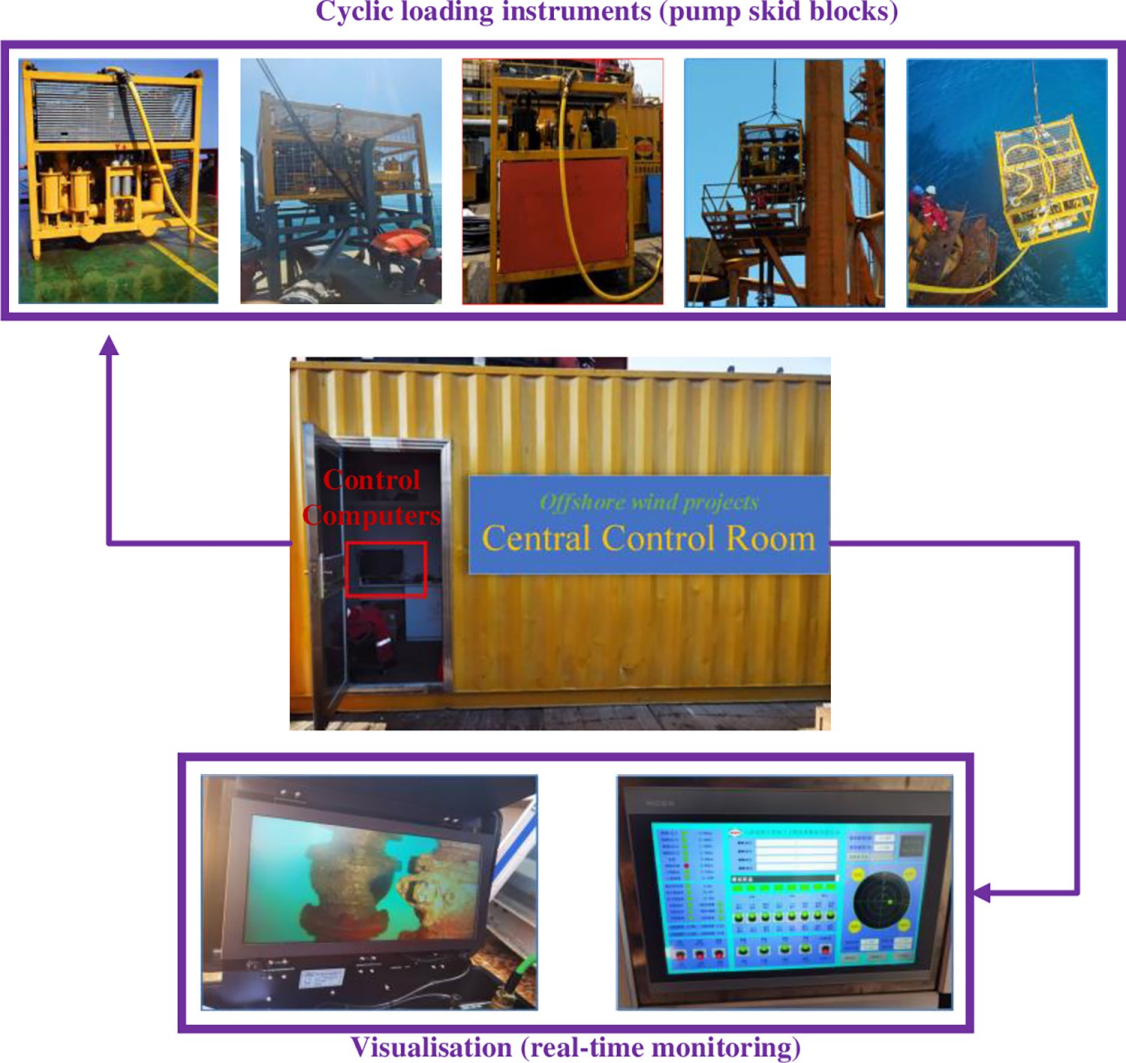
Cyclic loading control system.

**Fig 12:**
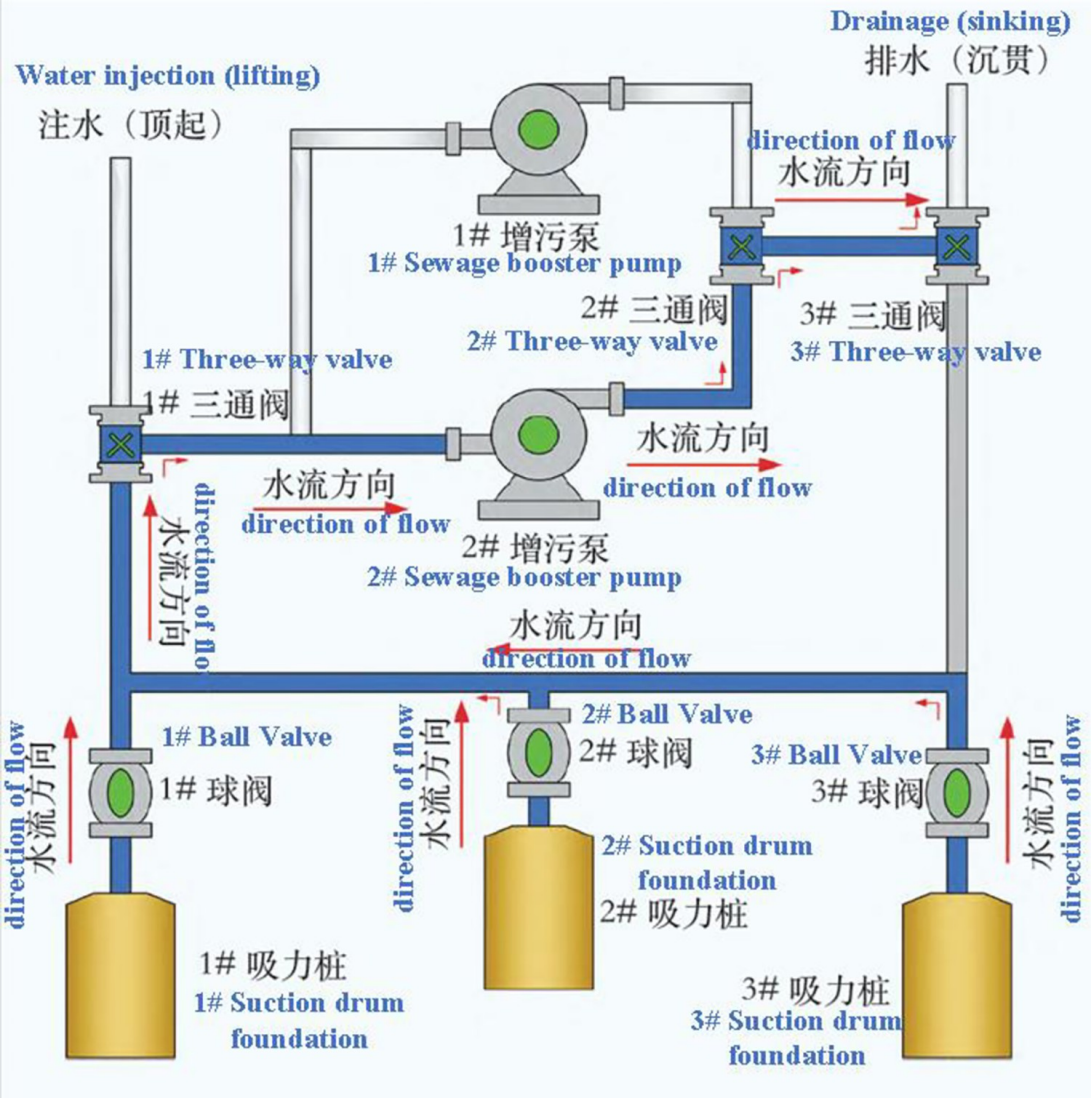
Cyclic loading workflow diagram.

The construction control instrumentation is detailed in Fig 11, and the cyclic loading workflow is shown in Fig 12. However, cyclic loading has certain limitations; if the suction force exceeds the upper limit suction force, the soil body will produce instability, resulting in the foundation sinking through the failure. At this time, the measures taken are to stop sinking through, using water injection jacking way to make the suction bucket foundation penetration into the soil body again, because in the construction, the actual allowable suction value is less than the theoretical allowable suction value, so it has not entirely reached the upper limit of suction at this time, so you can continue to increase the suction, improve the available penetration force.

### 4.2. A comparative analysis of negative pressure sinking

**Fig 13:**
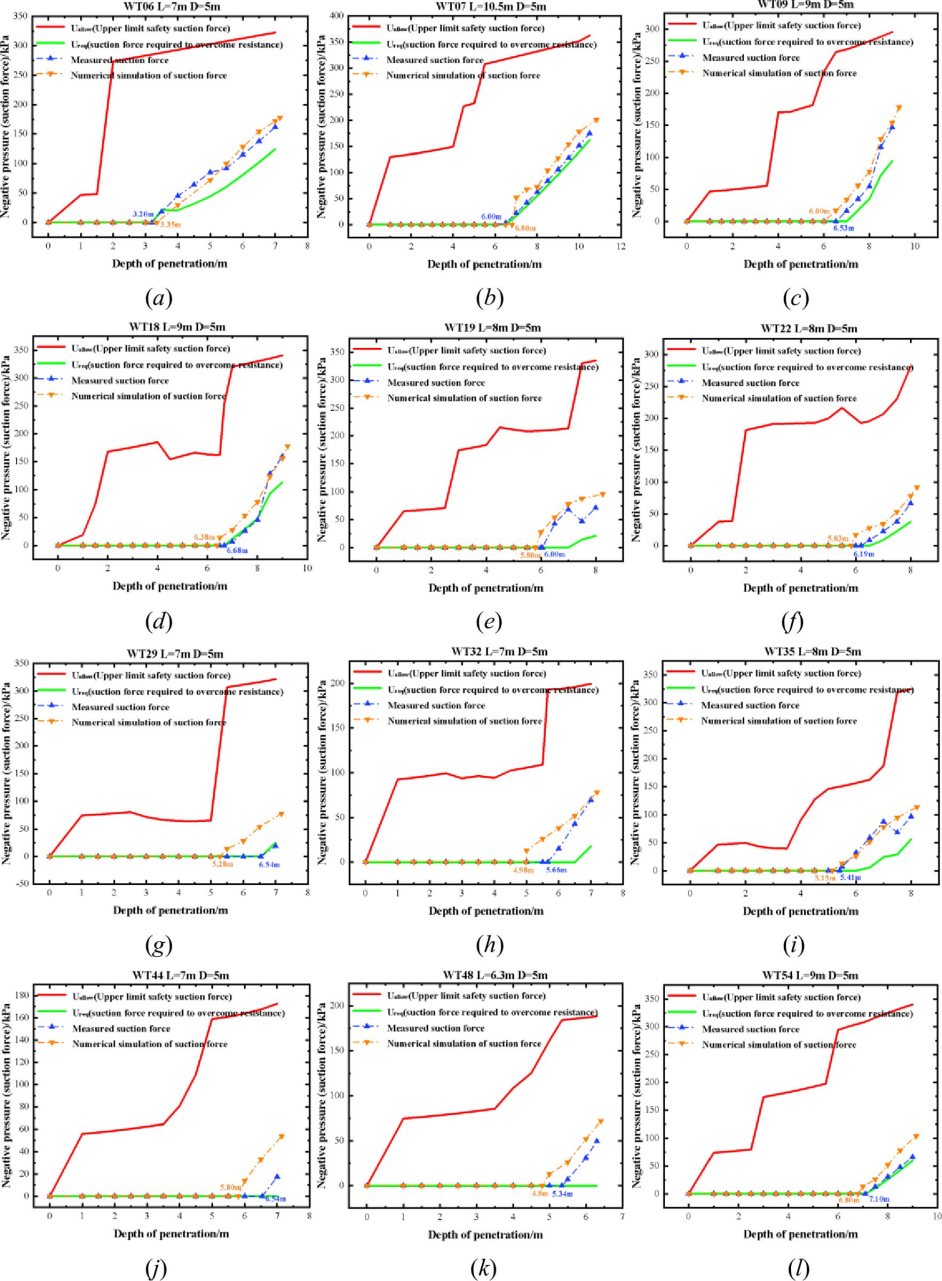
Comparison of negative pressure (suction) and sinking depth curves for the WT06, WT07, WT09, WT18, WT19, WT22, WT29, WT32, WT35, WT44, WT48, and WT54 bases.

To assess the validity of the finite element simulation of negative pressure sinking penetration on the suction drum foundation and the theoretical predictions of negative pressure penetration, we initially analyzed twelve suction drum foundations alongside the theoretical suction analysis prediction. Subsequently, we compared the negative pressure penetration depth of the suction drum foundation, obtained via measured negative pressure data during the construction process, with the finite element simulation of the suction drum under negative pressure penetration and the theoretical calculation of the suction drum under negative pressure penetration depth. The outcomes, presented in Fig 13(A)–13(l), clearly illustrate the results.

The negative-pressure penetration depth obtained through finite-element simulation, theoretical calculation, and construction measurement data (as depicted in Fig 13(A)–13(L)) shows a high degree of coherence. The difference between the self-weight penetration depth measured during construction and the depth simulated through finite element analysis is between 0.15m and 1.26m. Furthermore, the error difference between the self-weight penetration depth measured during construction and calculated theoretically is between 0m and 0.96m. Twelve machine positions (WT06 to WT54) are deemed safe for penetration throughout negative pressure sinking and penetration. Measured negative pressure values remain within the upper and lower safety limits during construction. The negative pressure measured during construction and the negative pressure from the finite element simulation exceed the theoretical minimum limit for safe suction (required suction) yet remain well below the maximum limit of safe suction (permissible suction). This redundancy factor guarantees the structure’s safety.

The contrast between the negative pressure penetration depth obtained via ABAQUS software FEM analysis and the measured negative pressure penetration depth data mainly arises from soil property factors and suction drum foundation load constraints. (1) The penetration principle of the suction drum foundation is specific. Therefore, the finite element analysis did not consider the effect of seepage and changes in pore water pressure on the penetration resistance. As a result, differences in suction values were not taken into account. (2) In actual construction, the contact between the suction drum foundation and the soil is subject to temporal variations. At the same time, the finite element simulation only permits choosing a contact mode that does not separate the two but allows for tangential sliding.

### 4.3. An analysis of the sinking resistance

Finite element simulation calculations can effectively predict the penetration resistance of the suction drum foundation to confirm the validation of theoretical calculations and verify the results’ reasonableness. Table 30 presents the compared and analyzed results of the suction drum foundation sinking resistance measured at twelve different positions (WT06, WT07, WT09, WT18, WT19, WT22, WT29, WT32, WT35, WT44, WT48, WT54) in the construction project.

**Table 30:** Comparative analysis of theoretical and finite element and measured penetration resistance.

Suction Barrel Foundation	Theoretical *Q*_tot_/(kN)	FEM *Q*_tot_/(kN)	Measured *Q*_tot_/(kN)	Measured and Theoretical deviation values/(kN)	Measured and FEM deviation values/(kN)
WT06	4841.19	5379.61	4900.29	59.1	-479.32
WT07	5707.28	5916.94	5101.05	-606.23	-815.89
WT09	4326.49	4541.23	4693.66	367.17	152.43
WT18	4702.80	4890.55	4873.21	170.41	-17.34
WT19	3297.08	3768.72	3640.45	343.37	-128.27
WT22	3641.83	3611.21	3549.13	-92.7	-62.08
WT29	2891.10	3291.11	2936.61	45.51	-354.50
WT32	2746.48	2755.35	2445.21	-301.27	-301.14
WT35	3540.25	4138.91	4014.82	474.57	-124.09
WT44	2209.50	2498.69	2625.12	415.62	126.43
WT48	1930.01	2260.80	2067.48	137.47	-193.32
WT54	3645.07	3673.80	3579.96	-65.11	-93.84

The table shows a slight difference between the theoretical, finite element simulation, and measured values. The reasons for the error are: (1) The theoretical calculation method relies on engineering experience and does not have a quantitative analysis of the theoretical model. This method is not necessarily suitable for soil layers with different geologies, and the difference between each parameter leads to a difference between the measured and theoretical values of total resistance for sinking penetration. (2) The difference between the measured and finite element simulation values is due to the soil body disturbance during the suction bucket foundation sinking process, which needs to be considered when building the model. The seepage of sand and soil, as well as the constraints and loads of the model, did not align precisely with the load conditions of the actual construction, which led to a certain amount of error. Nevertheless, the API specification’s theoretical calculation and ABAQUS software’s FEM analysis can predict suction drum foundation sinking resistance more accurately.

The table above shows that the suction drum foundation with the same drum length has varying penetration resistance in different soil layers. Moreover, the total resistance of the suction drum foundation in sandy soil is less than that in clay soil. Hence, it can be inferred that the sandy soil reduces the penetration of the suction drum foundation under the suction mode.

## 5. Conclusions

This study systematically investigates the sinking characteristics of suction drum foundations based on measured data from the YueDian Yangjiang Shapa offshore wind power project, using various technical methods, including theoretical analysis, numerical simulation, and data mining. The study analyzed the feasibility of the suction drum foundation sinking and its suction sinking and resistance. Furthermore, the design theory of suction drum foundations in geotechnical engineering was summarised, and the theoretical foundation for studying its engineering characteristics and developing engineering technology was established. Finally, the study provides a preliminary suction drum foundation design reference and concludes with its main findings.

(1) The measured data of the suction drum foundation sinking processes for WT06, WT07, WT09, WT18, WT19, WT22, WT29, WT32, WT35, WT44, WT48, and WT54 were collected, collated, and analyzed. The data were compared to theoretical calculations and finite element simulations according to API specifications. The results confirm the accuracy of the theoretical and finite element simulation analyses consistent with API specification. The results of theoretical and finite element simulation analyses according to API specification and the actual suction drum foundation construction are consistent with the measured suction force and sinking resistance values. This confirms the safety, reliability, and accuracy of the values derived from theoretical analyses and simulation calculations.

(2) The API theoretical formula is suitable for calculating the penetration resistance of the suction drum foundation. The most critical physico-mechanical indices impacting the suction drum foundation penetration resistance are cohesion, internal friction angle, and effective gravity. They can accurately forecast the penetration resistance and provide a significant theoretical foundation for designing, calculating, and assessing the construction risks of suction drum foundations.

(3) There is a deviation in the results obtained from the ABAQUS finite element analysis, API specification analysis, calculation results, and field-measured data. The main reasons behind this deviation are: the influence of seepage and changes in pore water pressure on the penetration resistance has not been considered, which results in differences in suction values; the finite element calculation does not account for the influence of soil plugs on the sinking penetration characteristics due to slow loading of the foundation leading to differences in suction values; the mesh division during modeling, the constraints, and load settings used during calculation and analysis are not entirely consistent with the constraints and load conditions on-site. In modeling, the mesh division, constraints, and load settings are only partially consistent with the constraints and load conditions present during field construction.

(4) In the calculation of the construction design, the suction value of the foundation of the suction drum should be controlled within the range of safe suction limits, namely the minimum safe suction (required suction) and the maximum safe suction (permitted suction). If the suction value exceeds the required suction level, it can provide sufficient bearing capacity to resist sinking resistance. On the other hand, keeping the value below the permissible suction level can effectively prevent the occurrence of soil plugging in the barrel.

(5) On-site engineering measurement data is lacking for the suction drum foundation in layered soil. This study addresses this gap relying on field engineering measured data to fill the data void in layered soil sinking. It also provides relevant data to compare and correct indoor model test results with measured data, thus providing an excellent engineering reference value.

Limitations of Study. This article solely examines the temporary foundation guiding frame platform suction drum foundation, excluding the impact of wind and wave load. Further research will expand to include the permanent suction drum foundation concerning offshore wind power foundation and wave protection–with increased complexity in design and construction. The ALE method is utilized for numerical simulation to simulate the sinking of suction drum foundations. However, the method omits the role of soil seepage under certain assumptions. Therefore, future research will focus on how to achieve soil-water coupling.

## Supporting information

S1 File (ZIP)
