# Supplementary material for: Comparative analytical study of suction drum foundation penetration characteristics of guide frame platforms with real measurements
Source: PLoS One. 2024 Feb 29;19(2):e0299647. doi: 10.1371/journal.pone.0299647 (PMC10903861; doi:10.1371/journal.pone.0299647)
Supplement: S1 File — (ZIP) [file pone.0299647.s001.zip › Supporting Information/minimal data.docx]

**Proofs of conclusion 1**

Table 1 and Figure 1 confirm that the theoretical and finite element simulation analysis results of the API specification and the results of the actual suction force and sinking penetration resistance required for the construction of the suction drum foundation are highly compatible with the real suction force and sinking penetration resistance results, which confirms that the values derived from the theoretical analyses and simulation calculations are safe, reliable and have a high degree of fit.

**Table.1** Measured data and comparative analysis with API specification theoretical calculations and finite element simulations.

| Suction Barrel Foundation | Theoretical *Q*_tot_/(kN) | FEM *Q*_tot_/(kN) | Measured *Q*_tot_/(kN) | Measured and Theoretical deviation values/(kN) | Measured and FEM deviation values/(kN) |
| --- | --- | --- | --- | --- | --- |
| WT06 | 4841.19 | 5379.61 | 4900.29 | 59.1 | -479.32 |
| WT07 | 5707.28 | 5916.94 | 5101.05 | -606.23 | -815.89 |
| WT09 | 4326.49 | 4541.23 | 4693.66 | 367.17 | 152.43 |
| WT18 | 4702.80 | 4890.55 | 4873.21 | 170.41 | -17.34 |
| WT19 | 3297.08 | 3768.72 | 3640.45 | 343.37 | -128.27 |
| WT22 | 3641.83 | 3611.21 | 3549.13 | -92.7 | -62.08 |
| WT29 | 2891.10 | 3291.11 | 2936.61 | 45.51 | -354.5 |
| WT32 | 2746.48 | 2755.35 | 2445.21 | -301.27 | -301.14 |
| WT35 | 3540.25 | 4138.91 | 4014.82 | 474.57 | -124.09 |
| WT44 | 2209.50 | 2498.69 | 2625.12 | 415.62 | 126.43 |
| WT48 | 1930.01 | 2260.80 | 2067.48 | 137.47 | -193.32 |
| WT54 | 3645.07 | 3673.80 | 3579.96 | -65.11 | -93.84 |


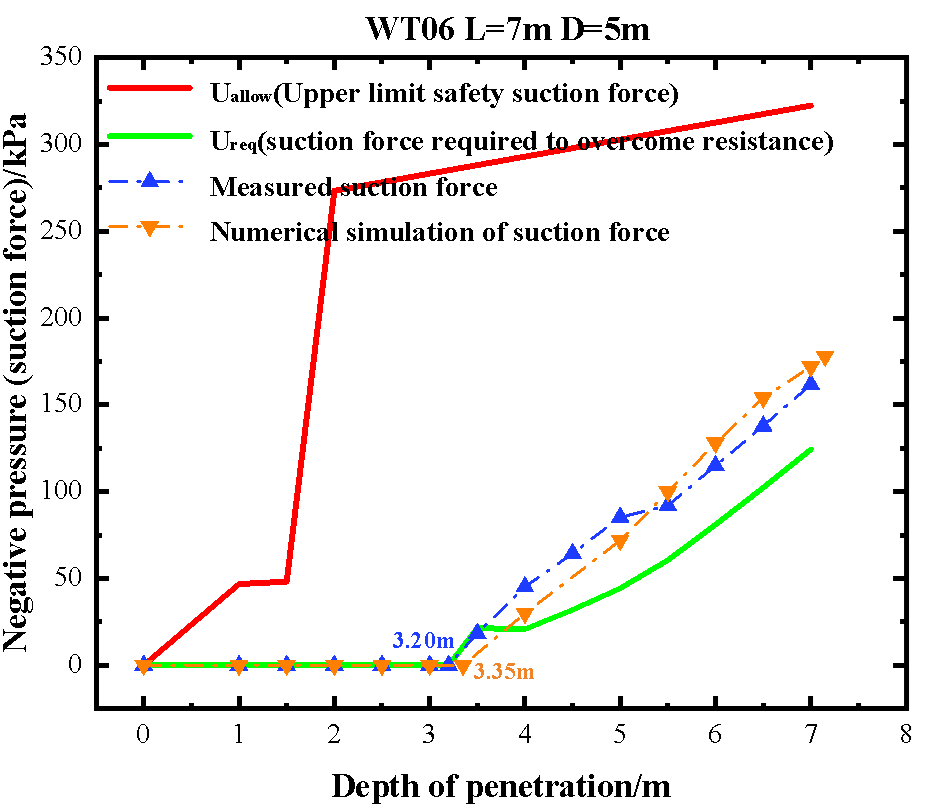

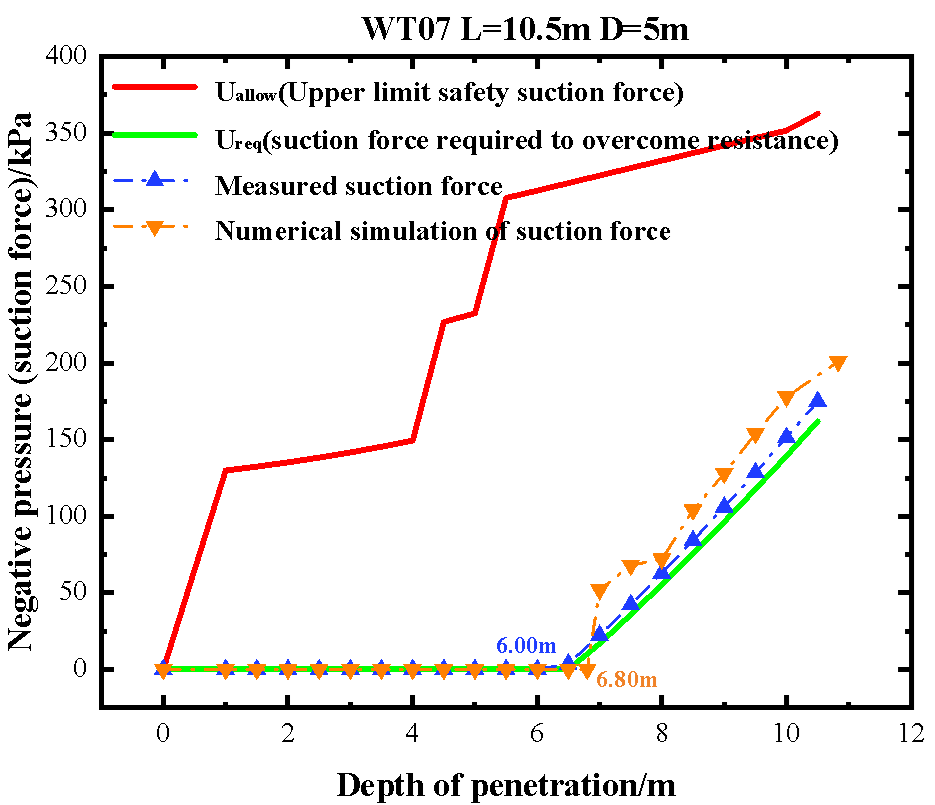


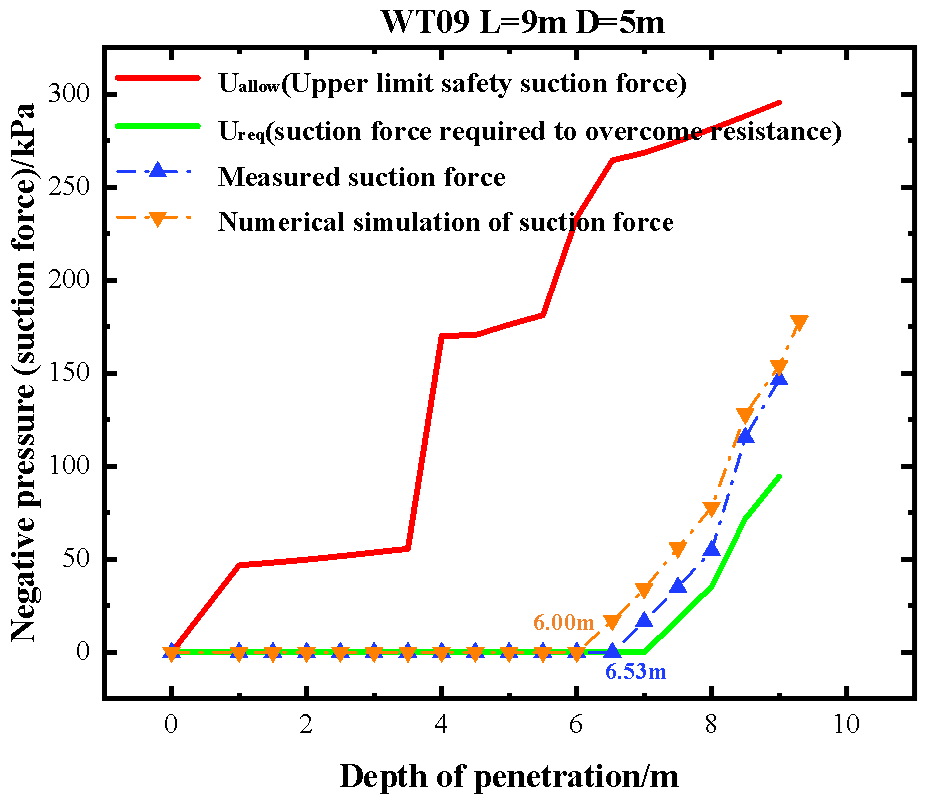

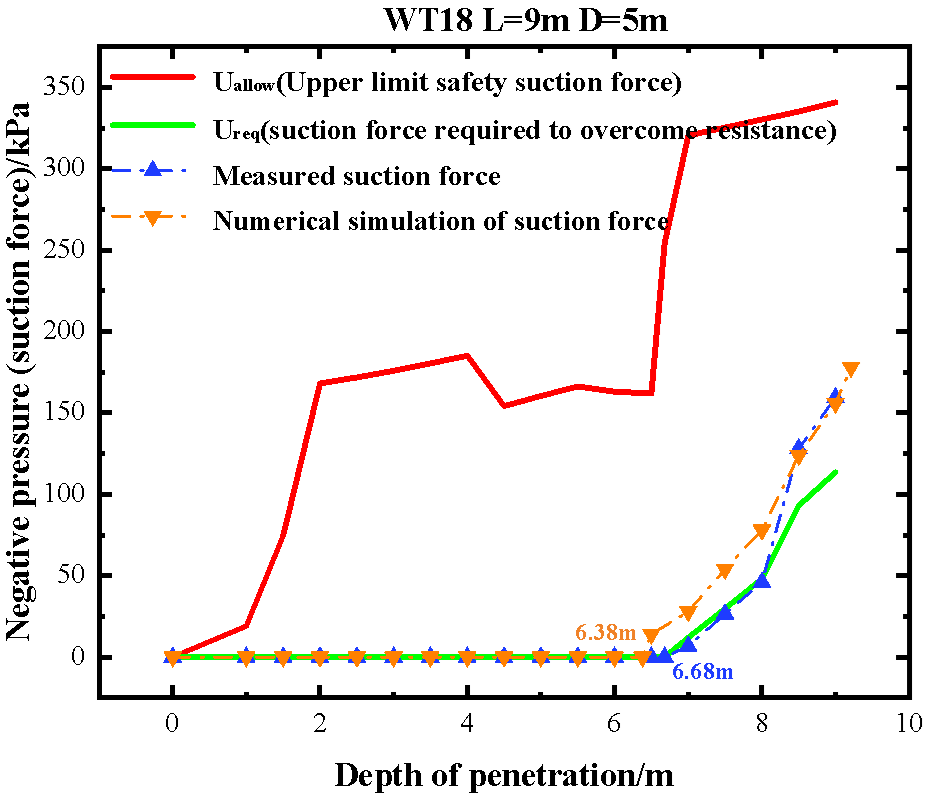


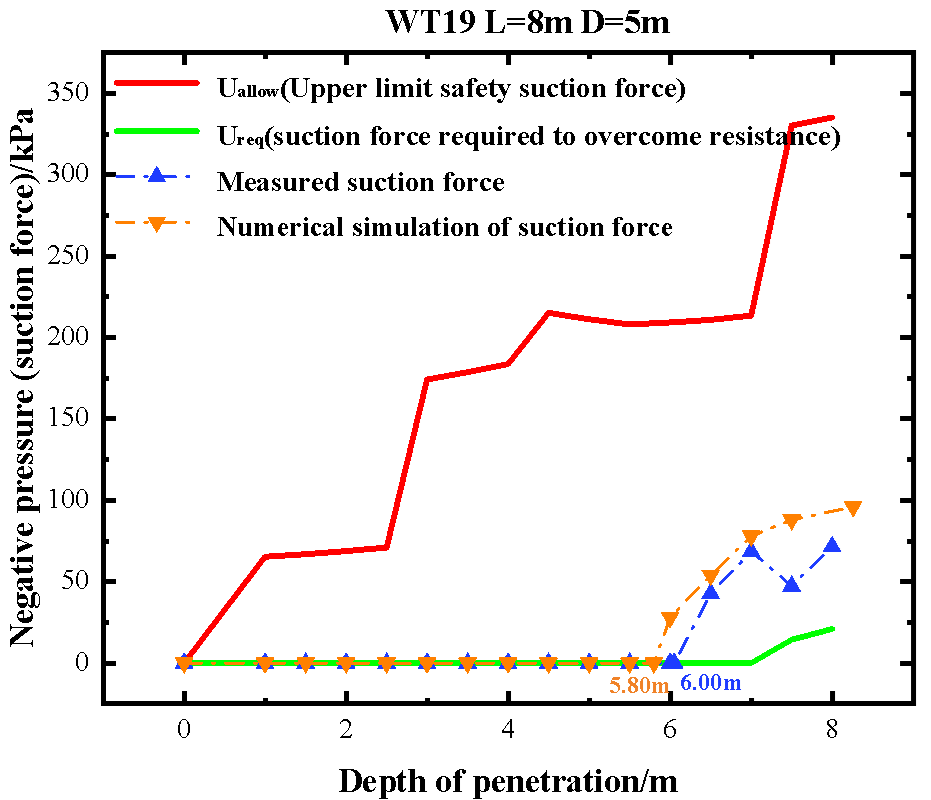

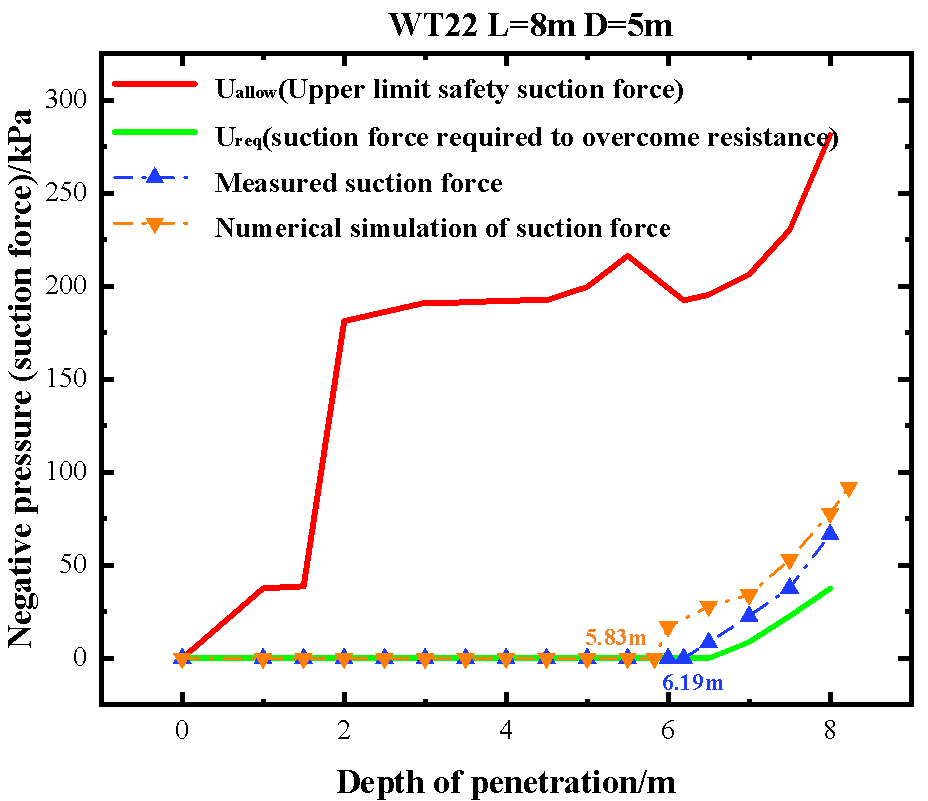


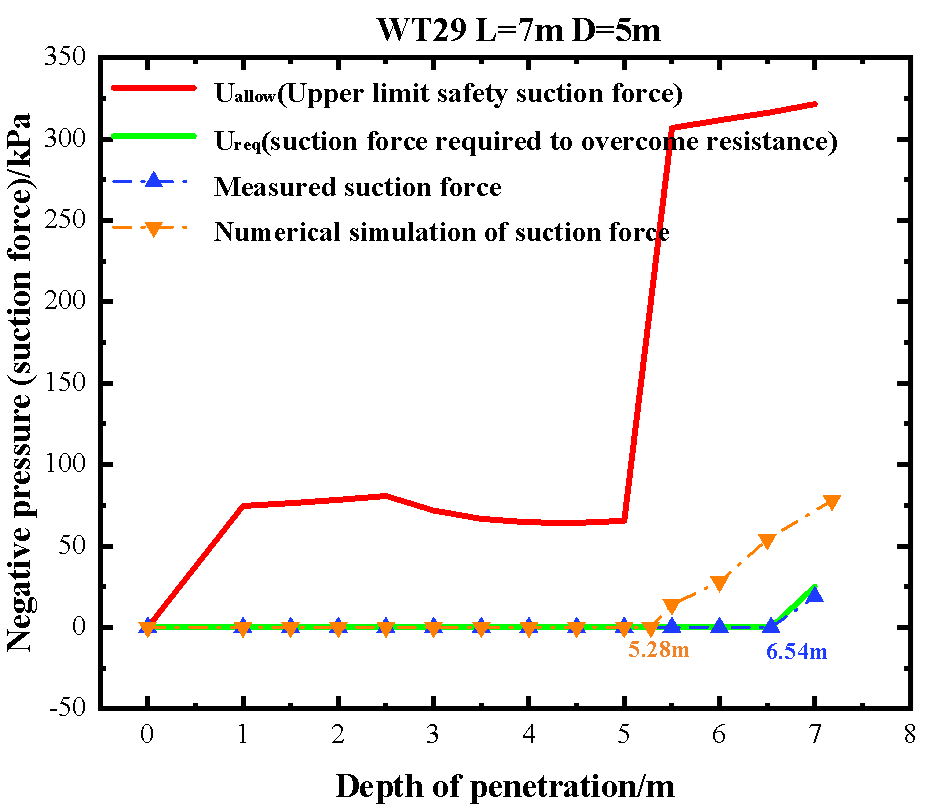

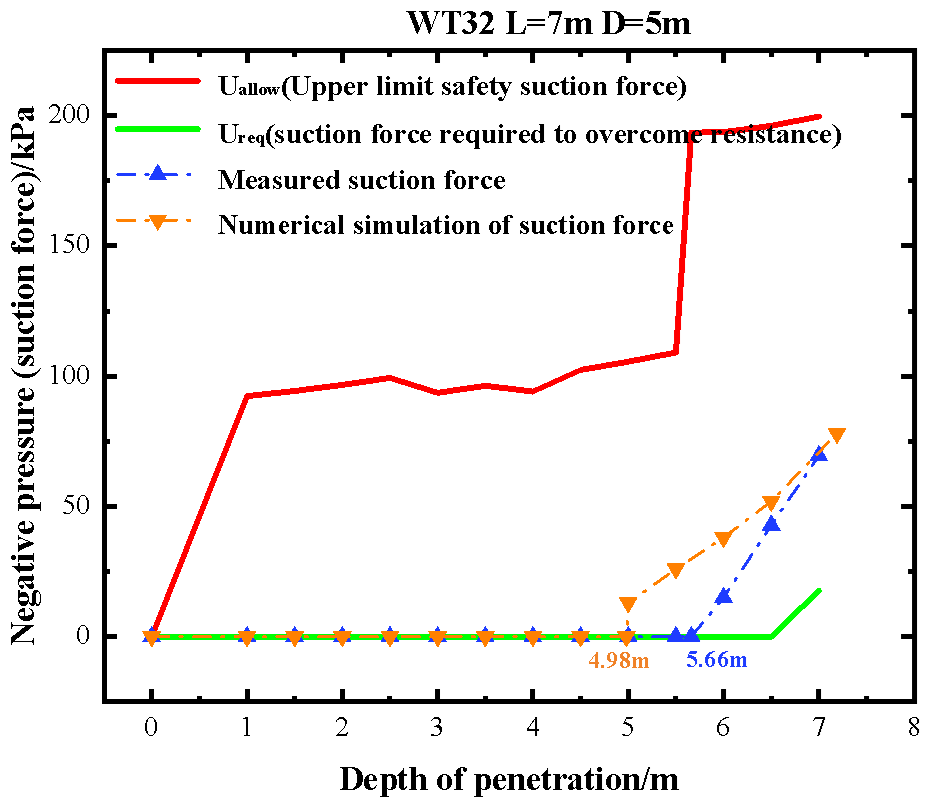


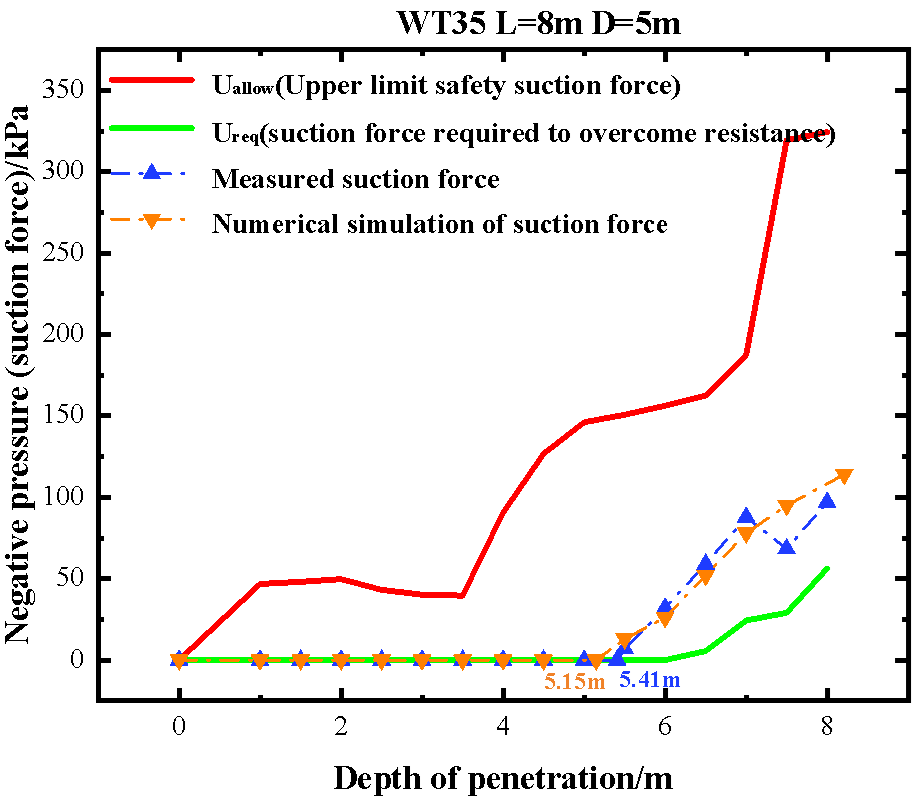

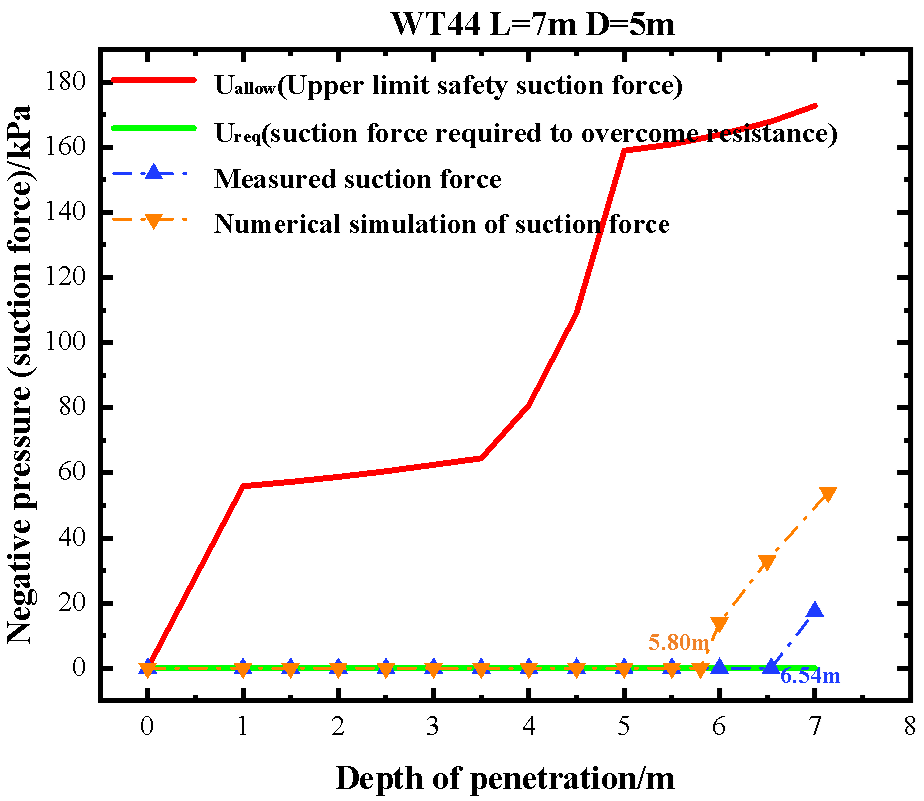


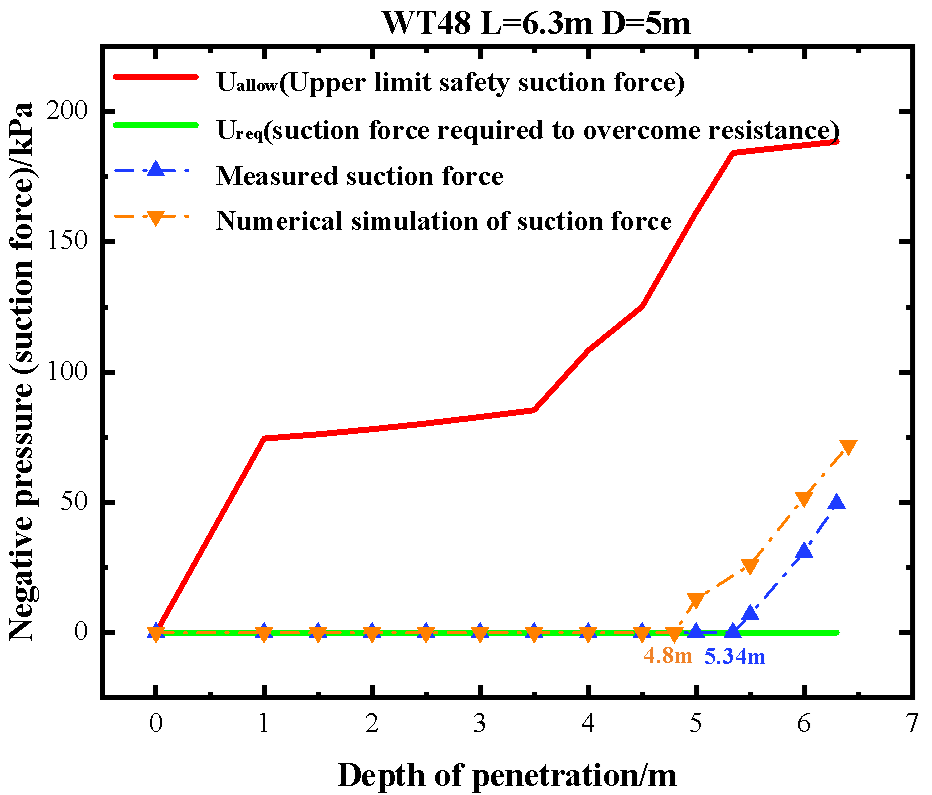

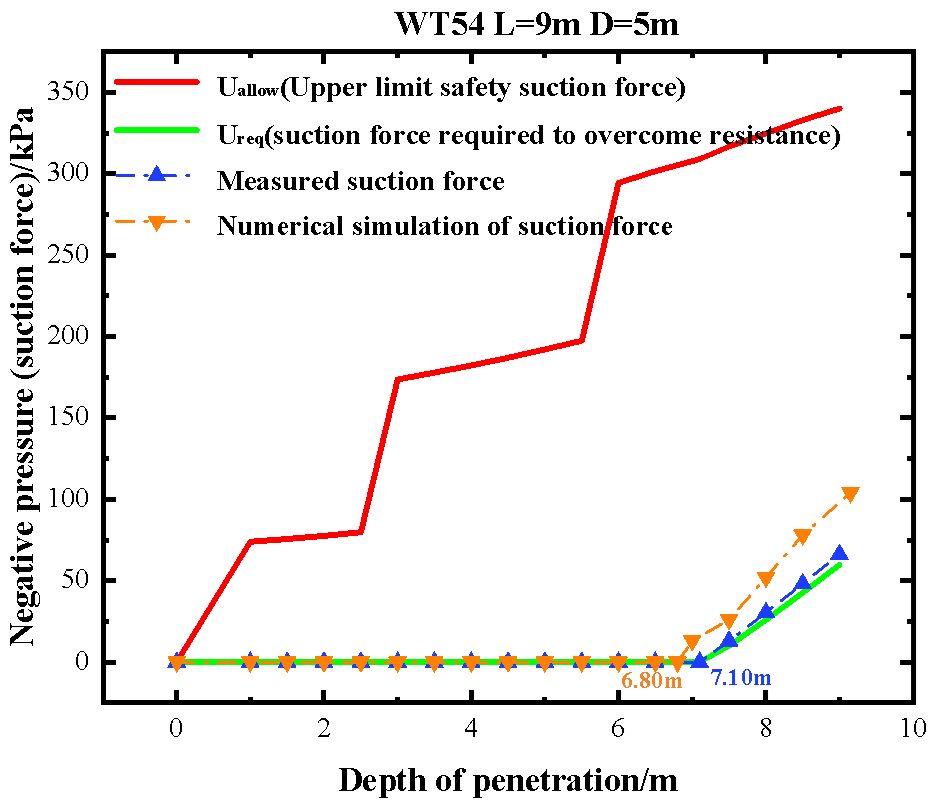


**Fig 1.** Negative pressure (suction) versus sinking depth curves for WT06, WT07, WT09, WT18, WT19, WT22, WT29, WT32, WT35, WT44, WT48, WT54 bases

**Proofs of conclusion 2**

The leading physico-mechanical indicators affecting the resistance to penetration of suction drum foundations are cohesion, angle of internal friction, and effective gravity. These indicators can effectively predict penetration resistance.

Understand the correlation between physical parameters such as water content, pore ratio liquidity index, and penetration resistance. Based on the data in the geotechnical investigation report, the correlation between the physical parameters and penetration resistance in the project area was analyzed, and the results are shown in Table 2 and Figure 2 (a) (b) (c).

**Table.2** Results of correlation analysis between physical parameters and penetration resistance

| independent variable | regression equation | $R^{2}$ | note |
| --- | --- | --- | --- |
| $w$ | $Q_{tot}=9.356w^{(-3.383)}$ | 0.847 | Figure 2 (a) |
| $e$ | $Q_{tot}=13188.532e^{(-2.707)}$ | 0.70 | Figure 2 (b) |
| $I_{L}$ | $Q_{tot}=5176.87IL^{\left(-1.945\right)}$ | 0.879 | Figure 2 (c) |


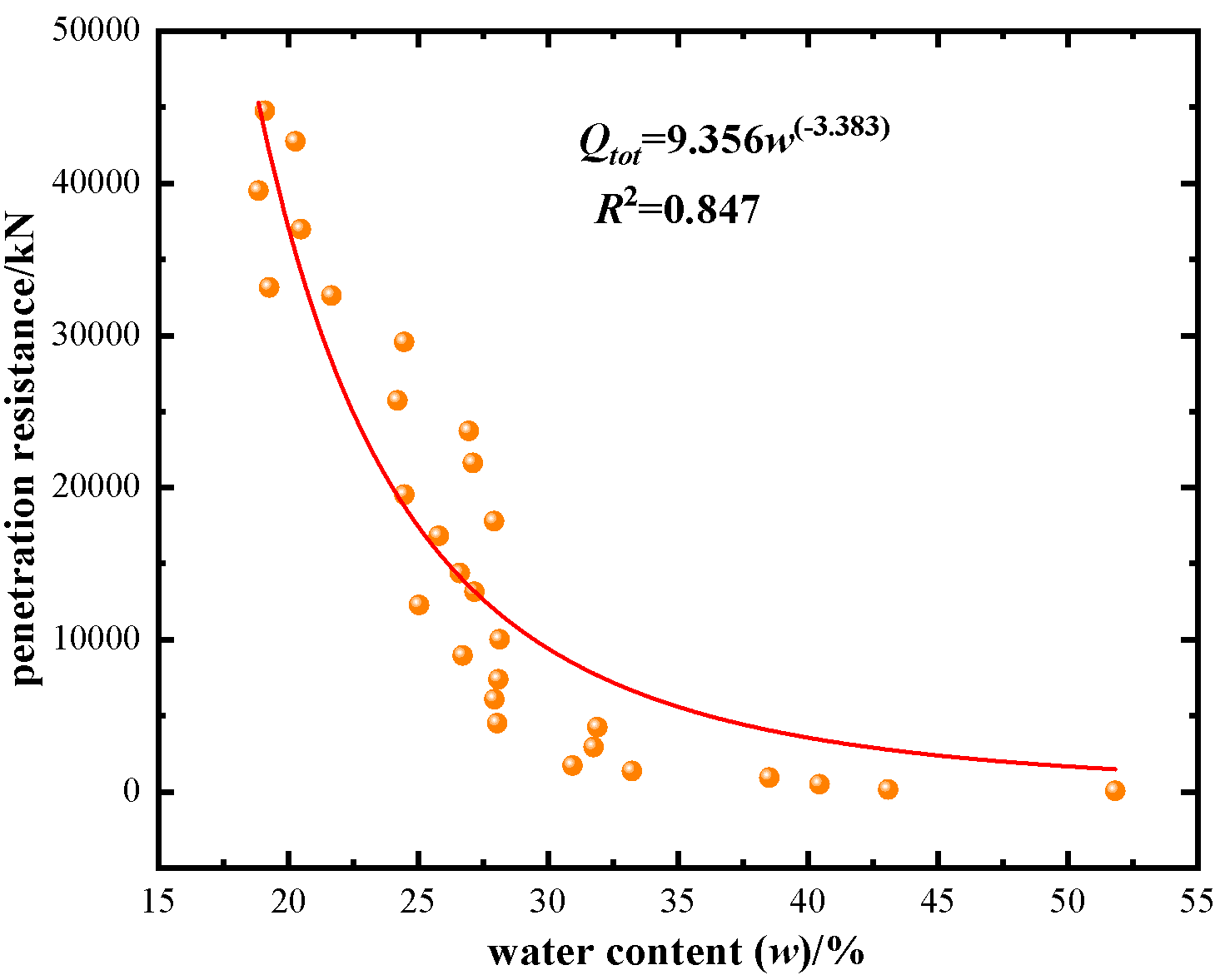

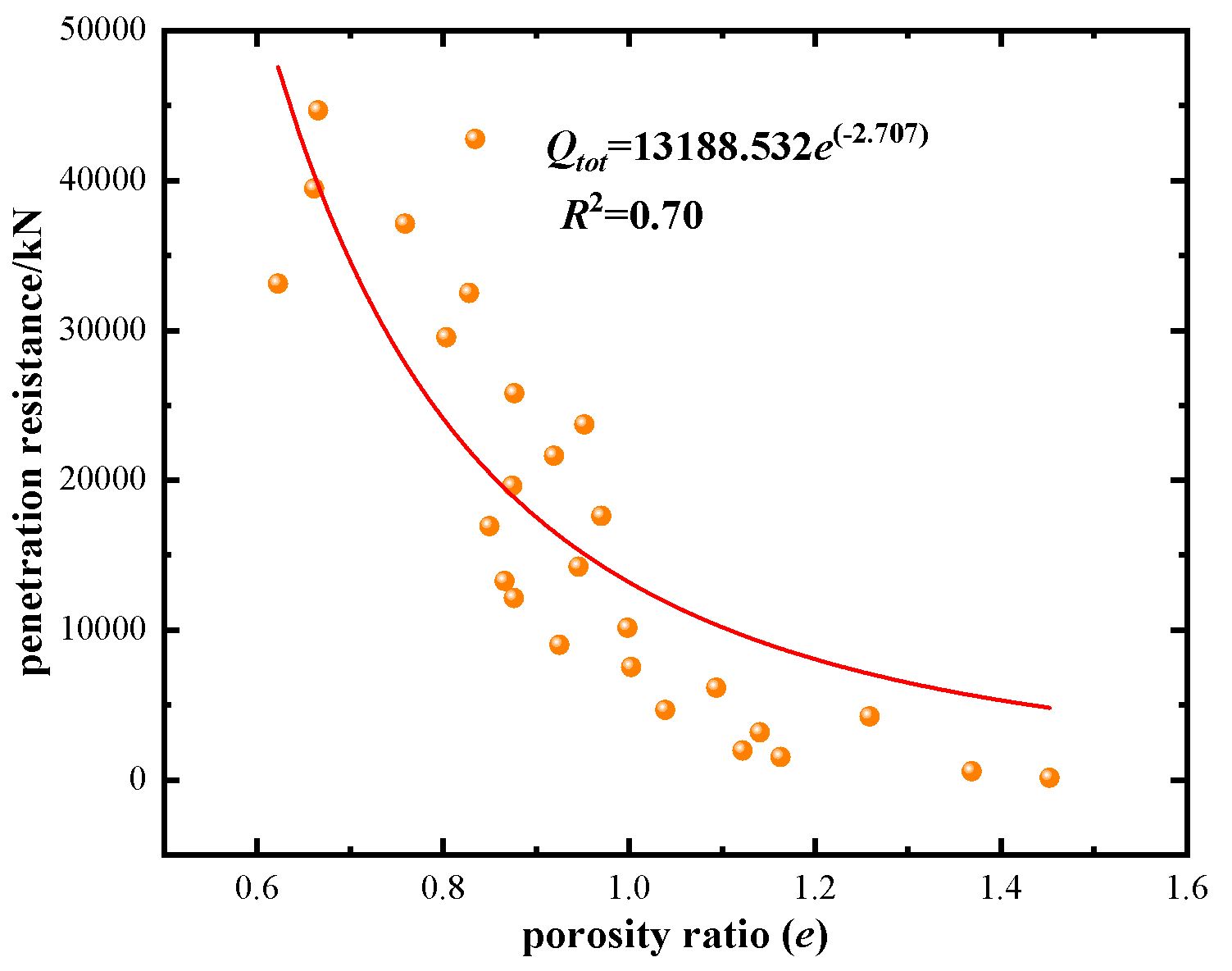


(a) (b)


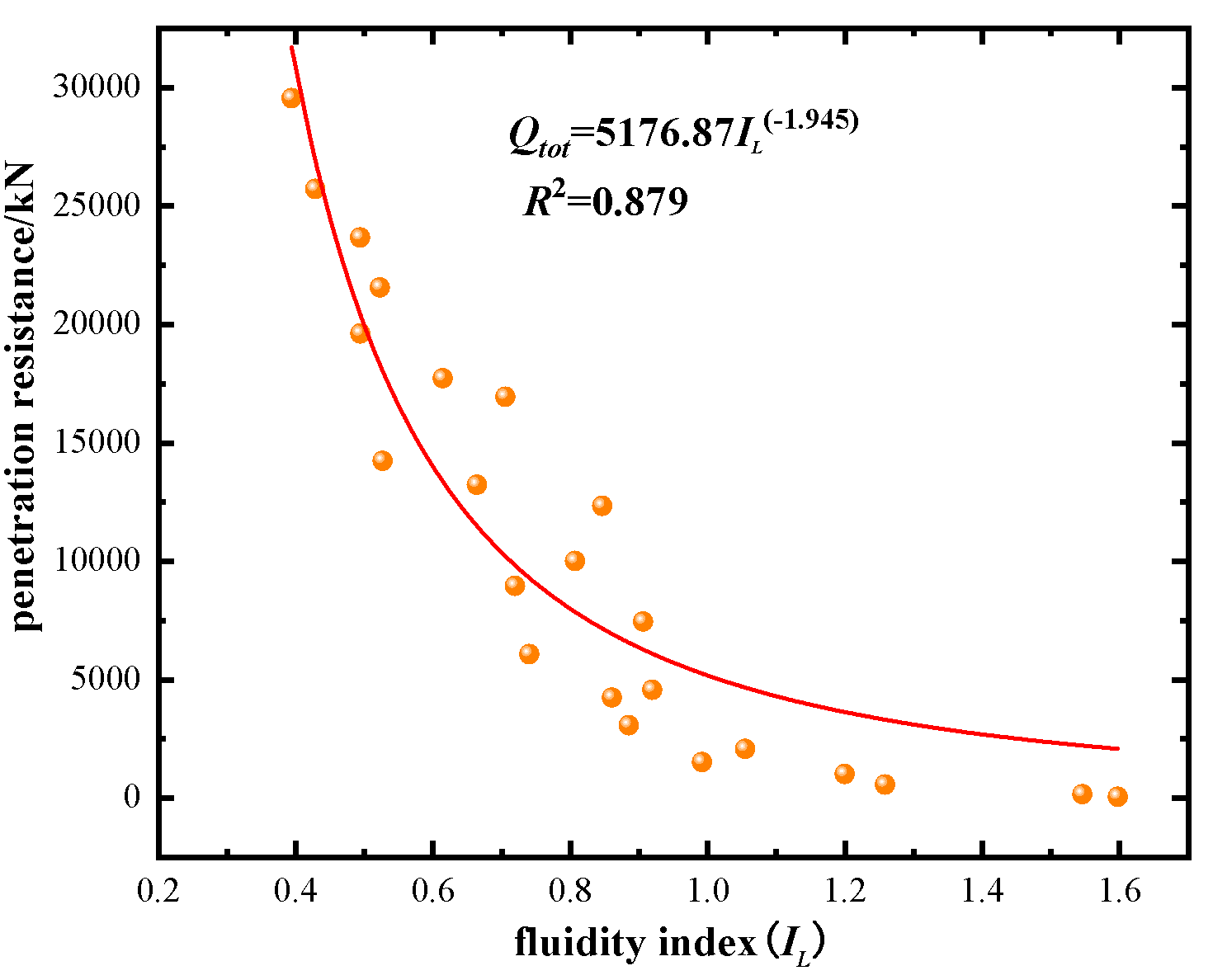


(c)

**Fig 2.** Physical parameters and penetration resistance curves


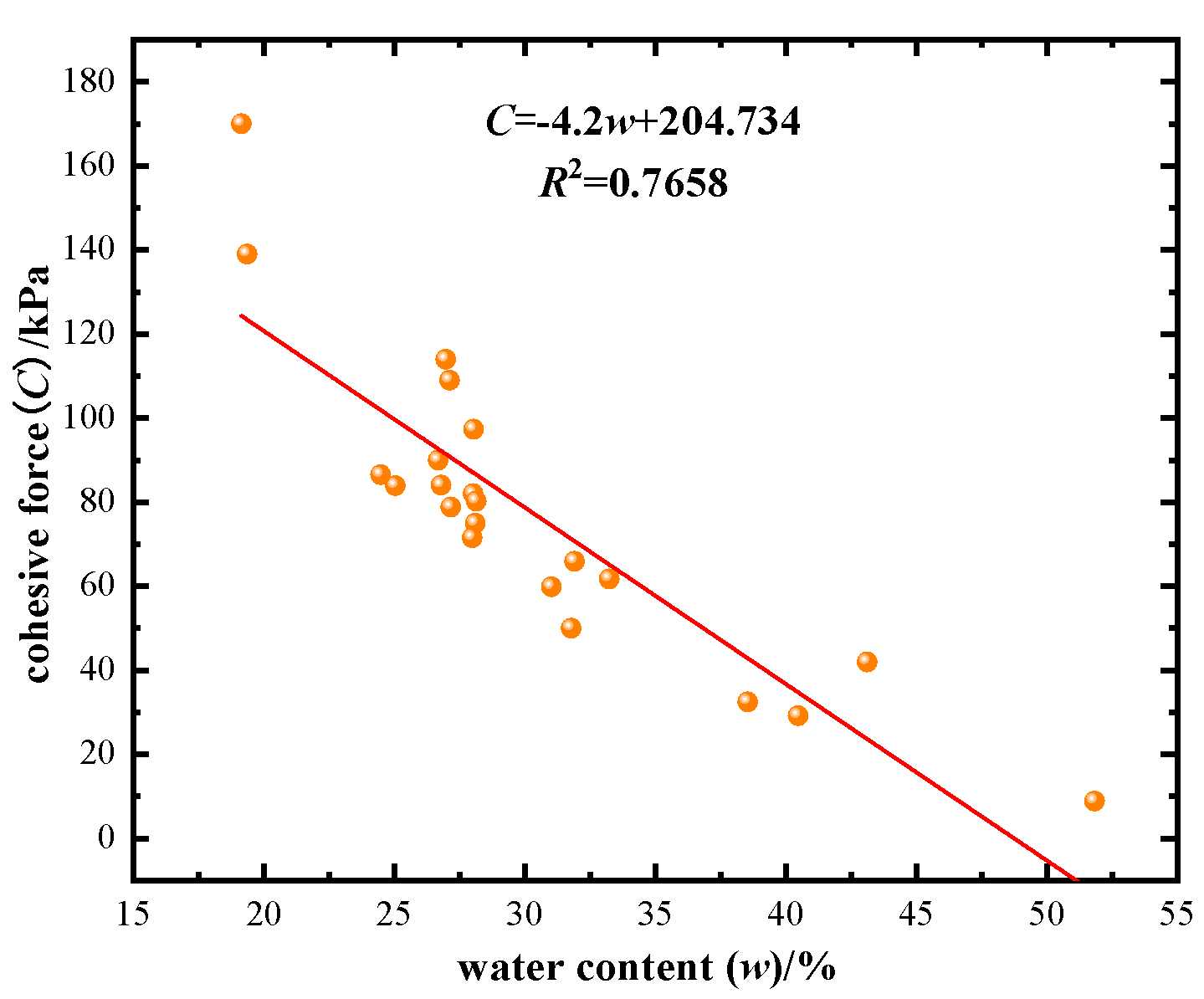

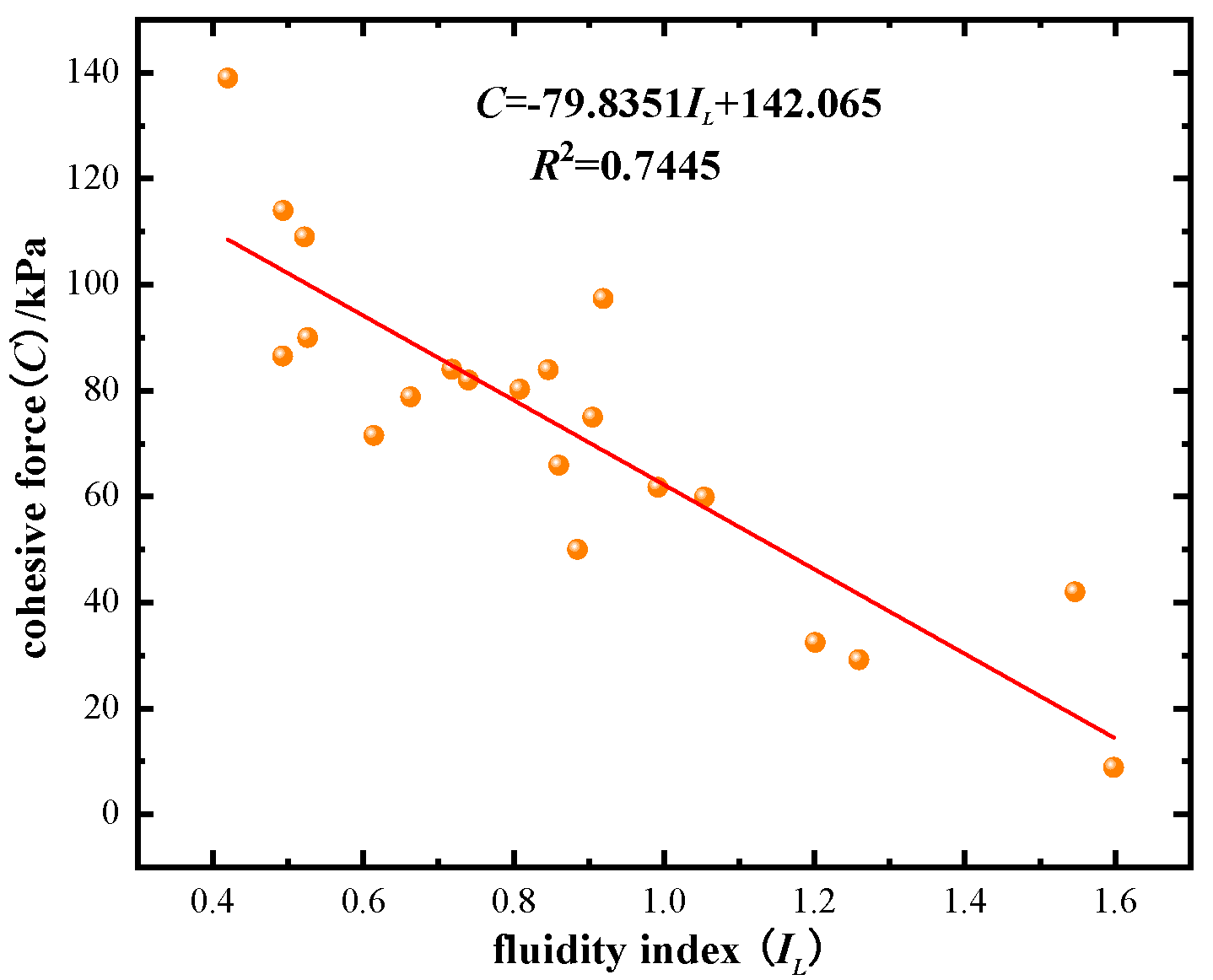


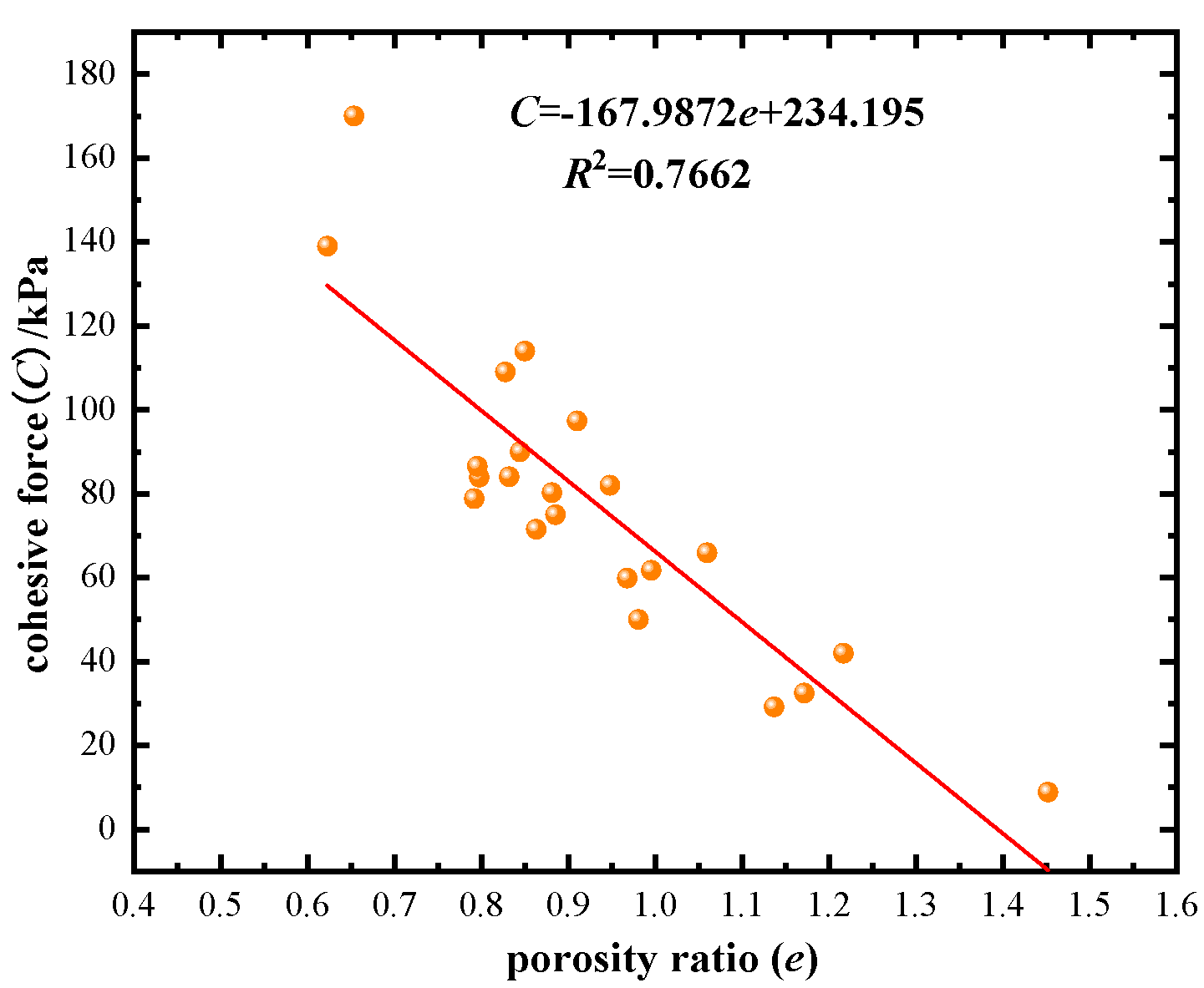


**Fig 3.** Curves of water content, pore ratio, liquidity index versus cohesive strength


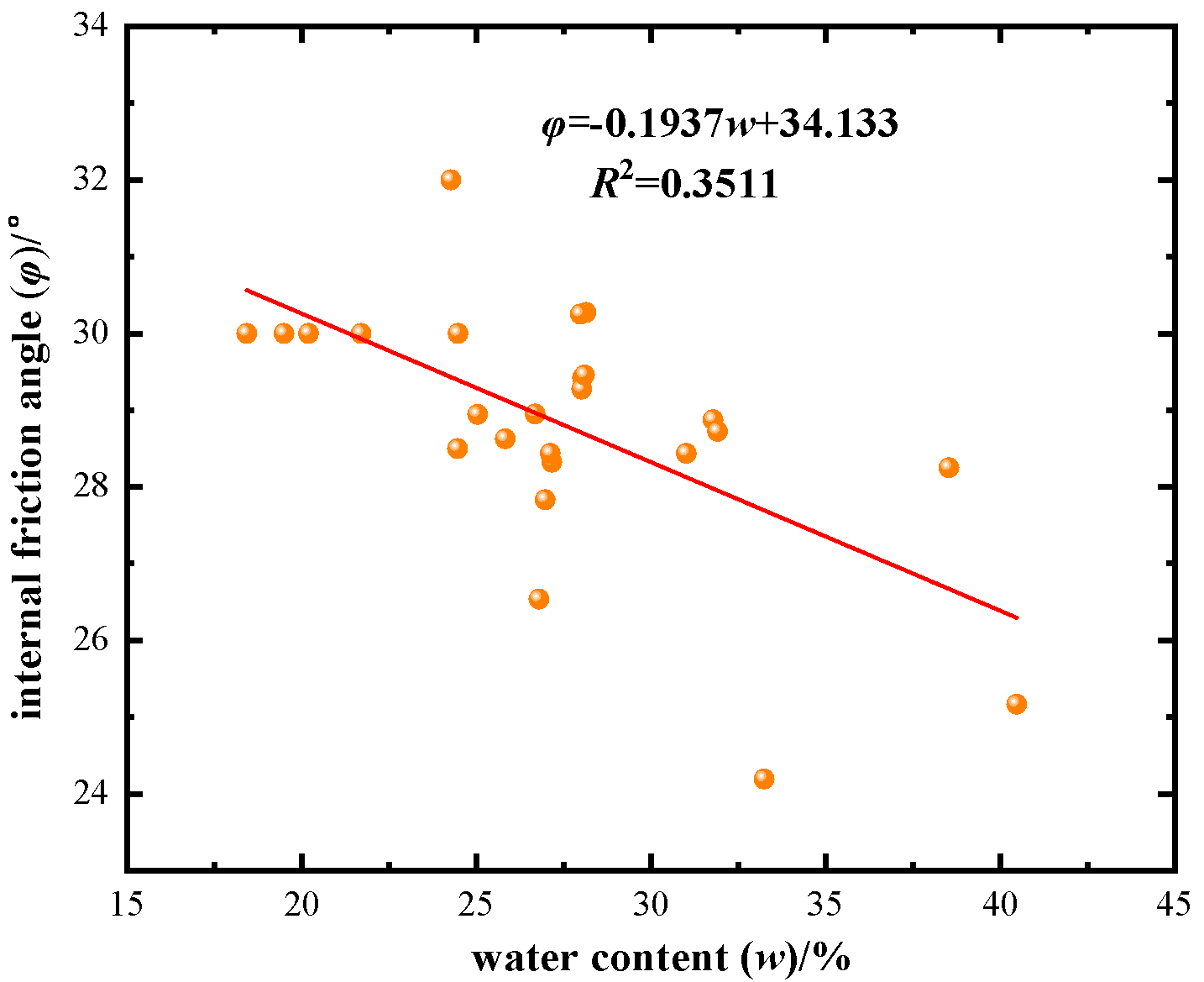

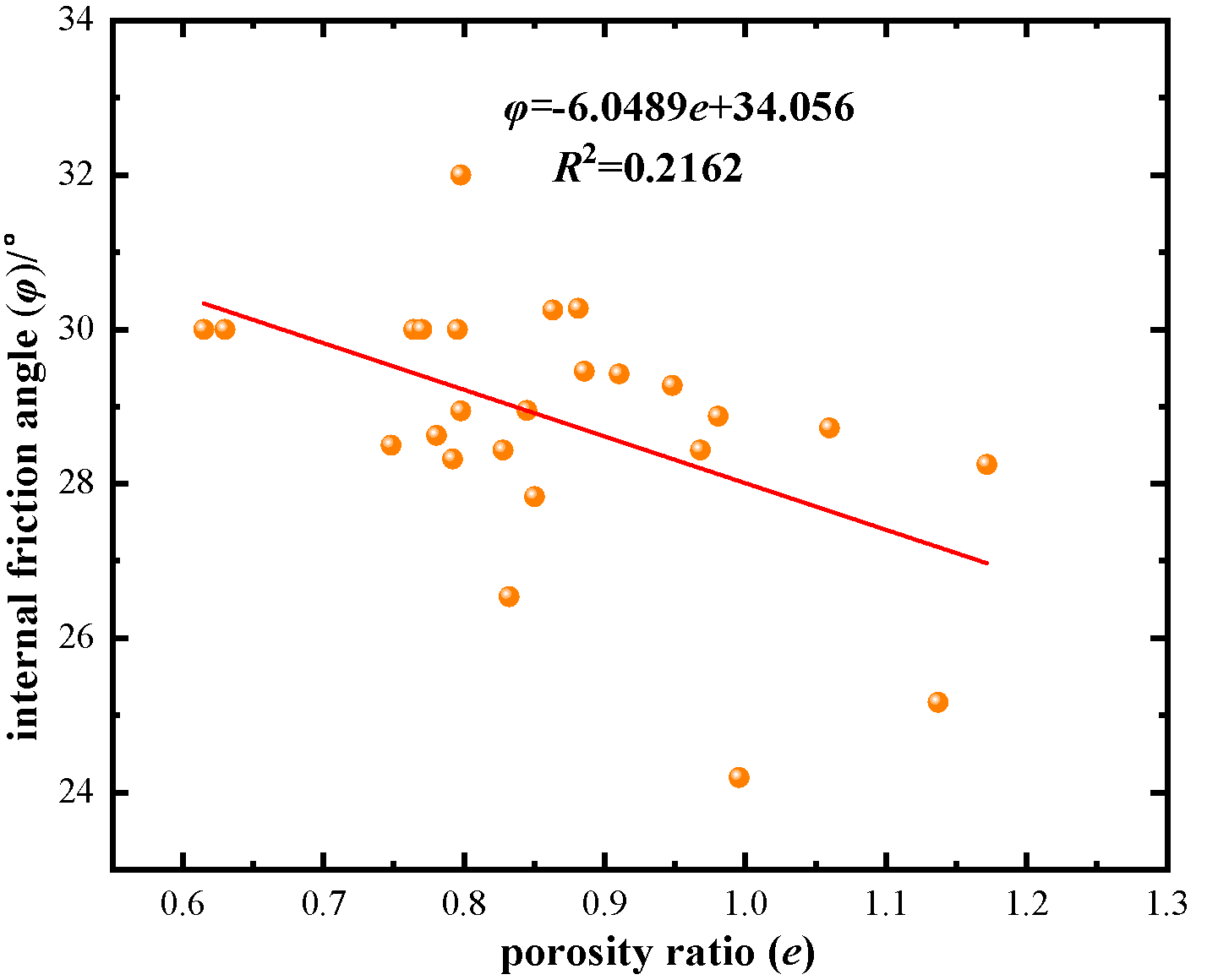


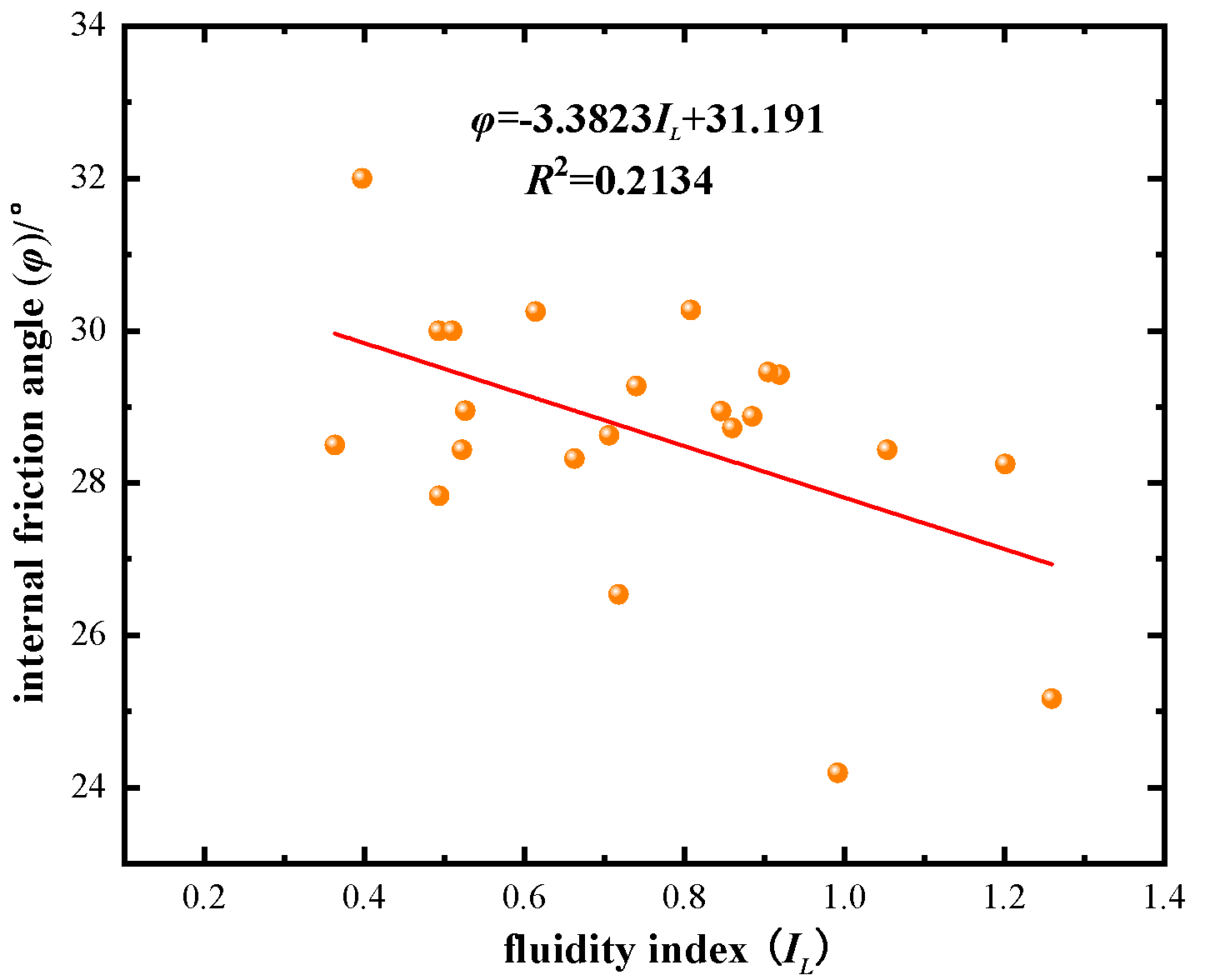


**Fig 4.** Curves of water content, pore ratio, liquidity index versus angle of internal friction
